# Retrospective Analysis of Autologous Chondrocyte-Based Cytotherapy Production for Clinical Use: GMP Process-Based Manufacturing Optimization in a Swiss University Hospital

**DOI:** 10.3390/cells11061016

**Published:** 2022-03-17

**Authors:** Virginie Philippe, Alexis Laurent, Nathalie Hirt-Burri, Philippe Abdel-Sayed, Corinne Scaletta, Valentine Schneebeli, Murielle Michetti, Jean-François Brunet, Lee Ann Applegate, Robin Martin

**Affiliations:** 1 Orthopedics and Traumatology Service, Lausanne University Hospital, University of Lausanne, CH-1011 Lausanne, Switzerland; valentine.schneebeli@unil.ch (V.S.); robin.martin@chuv.ch (R.M.); 2 Manufacturing Department, LAM Biotechnologies SA, CH-1066 Epalinges, Switzerland; alexis.laurent@lambiotechnologies.com; 3 Regenerative Therapy Unit, Plastic, Reconstructive and Hand Surgery Service, Lausanne University Hospital, University of Lausanne, CH-1066 Epalinges, Switzerland; nathalie.burri@chuv.ch (N.H.-B.); philippe.abdel-sayed@chuv.ch (P.A.-S.); corinne.scaletta@chuv.ch (C.S.); murielle.michetti@chuv.ch (M.M.); 4 DLL Bioengineering, Discovery Learning Program, STI School of Engineering, École Polytechnique Fédérale de Lausanne, CH-1015 Lausanne, Switzerland; 5 Cell Production Center, Service of Pharmacy, Lausanne University Hospital, University of Lausanne, CH-1066 Epalinges, Switzerland; jean-francois.brunet@chuv.ch; 6 Center for Applied Biotechnology and Molecular Medicine, University of Zurich, CH-8057 Zurich, Switzerland; 7 Oxford OSCAR Suzhou Center, Oxford University, Suzhou 215123, China

**Keywords:** ATMP, autologous chondrocyte implantation, cartilage defect, cell therapy, GMP manufacturing, optimization, process controls, production process, standardized transplant product, technical workflows

## Abstract

Cultured autologous human articular chondrocyte (HAC) implantation has been extensively investigated for safe and effective promotion of structural and functional restoration of knee cartilage lesions. HAC-based cytotherapeutic products for clinical use must be manufactured under an appropriate quality assurance system and follow good manufacturing practices (GMP). A prospective clinical trial is ongoing in the Lausanne University Hospital, where the HAC manufacturing processes have been implemented internally. Following laboratory development and in-house GMP transposition of HAC cell therapy manufacturing, a total of 47 patients have been treated to date. The main aim of the present study was to retrospectively analyze the available manufacturing records of the produced HAC-based cytotherapeutic products, outlining the inter-individual variability existing among the 47 patients regarding standardized transplant product preparation. These data were used to ameliorate and to ensure the continued high quality of cytotherapeutic care in view of further clinical investigations, based on the synthetic analyses of existing GMP records. Therefore, a renewed risk analysis-based process definition was performed, with specific focus set on process parameters, controls, targets, and acceptance criteria. Overall, high importance of the interdisciplinary collaboration and of the manufacturing process robustness was underlined, considering the high variability (i.e., quantitative, functional) existing between the treated patients and between the derived primary HAC cell types.

## 1. Introduction

The historical drive for the development and the clinical implementation of novel cell-based therapies has systematically been stronger and more diversified in university hospitals and in public hospital environments, whereas the registration and marketing of cell-based products are generally undertaken by private biotechnological industries [1,2,3,4]. Many European advanced therapy medicinal products (ATMPs) were classically developed within investigator-initiated clinical trials and were implemented into clinical practice before 2007, when majorly updated definitions and regulations notably entered into effect in the European Union (EU) [5,6,7,8,9]. Among the first established cell-based clinical therapeutic practices benefitting from the most scientific and medical hindsight are the applications of cultivated skin cells (e.g., stratified keratinocyte sheets) on severe burn patient wounds [10,11]. Parallelly, considerable research efforts and high clinical attention have been gathered, particularly around the field of stem cells, which were sourced, manufactured, and applied in various declinations for graft-versus-host disease, heart failure, Crohn’s disease, or liver, bone, and cartilage disfunctions, to cite only a few [12,13]. Despite the widespread and global uptake of such cell-based therapy and product use since the 1980s, many restricting and constraining quality-oriented regulations, similarly applied to public hospitals and to pharmaceutical industries, have drastically reduced the numbers of operational cell manufacturing facilities maintained in view of specific clinical applications [3,4,5,8,14]. Modern clinical implementations of cell-based treatments developed in university hospitals therefore currently rely on the availability of in-house cell production facilities complying with good manufacturing practices (GMP) and the related requirements or on the outsourcing of ATMP production via external contract manufacturing [3,8,15].

Despite the current scarcity of accredited GMP cell manufacturing platforms purposed with the continued bioengineering of skin tissues for burn victims, vast resources have been made available notably for the novel developments in autologous cytotherapeutic oncology applications (e.g., CAR-T cells) [16]. Due to the relatively high prevalence and to the vast diversity of the clinical affections within the oncology field, most of the recent technical advances with regard to clinical-grade cell therapy manufacture have been made around products designed for managing cancer in its various forms. Specifically, due to the autologous nature of many of these cell-based products, process-related adaptations have been made to the ad hoc manufacturing workflows, to optimally account for the short shelf-life and the volatile nature of the processed biological materials [16]. Due to burdening temporal and logistical constraints, several initiatives for point-of-care manufacturing of cell-based therapeutic products have emerged, which imply small-batch processing and multi-professional specific expertise [17]. Alternatively, several cost rationalization drivers have led to the emerging technological transition toward mobile infrastructures and automated bioreactor systems, for the insurance of both flexibility and efficiency of the GMP-compliant cell production [18,19,20,21]. All of these historical and current elements, taken together, have shown that modern cell-based therapeutic technologies require more proportionate ratios for risk and that there is a tangible need for pioneering regulatory developments, which should always be guided by the consolidated scientific and clinical experience accumulated to date [8,10]. However, the maintenance in operation of several accredited GMP platforms in university hospitals constitutes a prime testimony that historical practices can (and should) be perpetuated, notably in the domains of highly specialized regenerative medicine and oncology [1,8,16]. 

Alongside in vitro skin bioengineering, cartilage-based cell therapies benefit from vast historical hindsight in clinical settings and in industrial development for tissue-specific homologous and autologous applications [22,23,24,25,26]. The first reported in vivo experiments using in vitro cultures of articular chondrocytes, where the authors described the processes of primary cell isolation, expansion, and cryopreservation, were carried out by Smith et al. in rabbits and by Kawiak et al. in calves in the 1960s [27,28]. Such proceedings were soon adapted in view of developing novel therapeutic approaches in humans, by Manning and Bonner, who described the enzymatic cell dissociation process of chondrocytes from minced cartilage biopsies and their subsequent in vitro monolayer culture expansion using calf serum-supplemented proliferation medium [29]. We should note that the general process phases and the related technical specifications for chondrocyte in vitro isolation and culture, in view of therapeutic applications, have not substantially evolved since these first pioneering experiments, as the related research has merely been focused on the optimization of the tissue digestion solution composition or on the use of defined and serum-exempt cell culture medium [30,31,32,33,34]. Furthermore, the basic processes currently used for cultured chondrocyte clinical applications have been adapted from the works of Brittberg et al., which have served as a common basis for the development of several cell therapy products registered and marketed in the European or North American sectors (Table S1) [22,35].

Building on the available experience briefly mentioned hereabove, modern regenerative medicine approaches leveraging autologous chondrocyte implantation (ACI) classically comprise the use of human articular chondrocytes (HAC) as components of ATMPs or of combined ATMPs (cATMP) [22,23,24,25]. Therefore, the autologous cellular active pharmaceutical ingredients (API) may be obtained by ex vivo cellular amplification following the appropriate procurement and processing of a small healthy cartilage tissue biopsy (i.e., isolated from a part of the knee least exposed to mechanical stress) [22]. Then, in a second surgical step, the therapeutic cells (i.e., qualified as viable and chondrogenic) may be implanted as indicated for the repair of localized and symptomatic cartilage lesions of grades III or IV according to the ICRS classification [36]. By definition, the in vitro manufacture of cultured HAC-based cytotherapeutic products for human clinical use must be performed under an appropriate quality assurance system and following GMPs. In this context and based on the historical elements of ACI practice briefly mentioned herein, a prospective clinical trial was devised and is currently ongoing in the Lausanne University Hospital. Therefore, the HAC manufacturing processes have been iteratively optimized (e.g., use of pooled allogeneic human platelet lysate as a cell culture medium supplement) in the research laboratory and were then transposed for in-house GMP manufacture in view of investigational therapeutic use [37]. The highly encouraging preliminary clinical results and the full technical success of autologous HAC-based product provision for this clinical trial over the past four years enables a current robust assessment of the high quality of cytotherapeutic care provision, as presented herein. These critical advances were themselves founded and enabled by the effective multi-disciplinary collaboration and communication between the internal research and development, GMP manufacturing, and clinical orthopedic professional stakeholders within the Swiss institution. 

The general goal of this work was to propose, based on a retrospective analysis of the available clinical workflows and of the related GMP manufacturing records, an optimized process-based and parametric approach of HAC culture for therapeutic ACI, in order to outline some technical strengths and weaknesses and to provide renewed specific risk analysis-based solutions. The main specific aim of this study was therefore to retrospectively analyze clinical trial workflows, data compiled from the GMP manufacturing records, and data from certificates of the produced HAC-based investigational therapeutic products (i.e., primary cell types derived from 47 patient biopsies). This was performed in order to establish a renewed and detailed technical overview of the various steps and parameters entailed within the manufacture of HAC-based standardized transplant products (TrSt) in a Swiss university hospital setting. We were able to use all of the produced data to evaluate and to ameliorate parametric processes for the considered therapeutic materials, based on the analysis of existing and synthesized GMP records and data, in view of augmenting the overall quality of processes and of materials for further ACI-related clinical studies. This was performed notably through an industrial methodological approach, using renewed risk analysis-based process definition, with specific focus set on process parameters, controls, targets, and acceptance criteria. Overall, the existing in-house para-clinical research and technical experience pertaining to or used in support of HAC-based cell therapy product GMP manufacture were leveraged in this study. The presented results and considerations foremost underlined the high importance of multi-disciplinary collaboration and general manufacturing process robustness, considering the high variability (i.e., quantitative, functional) existing between patients and between the derived primary HAC cell types.

## 2. Materials and Methods

### 2.1. Materials Available for Retrospective Analysis and Ethical Compliance of the Study

#### 2.1.1. Clinical Trial Orthopedic Patient Files and Related GMP Manufacturing Records Analyses

This study was performed using materials, data, and information gathered in the context of an authorized prospective clinical trial (i.e., ClinicalTrials.gov Identifier: NCT04296487, “Introduction of ACI for Cartilage Repair”). This pilot monocentric clinical trial was initiated in 2017 and is currently ongoing, where at least 47 patients have received the HAC-based cell therapy in the form of ACI for traumatic focal chondral or osteochondral lesions of the knee. The data acquisition for the study was performed by compilation of patient medical files, performed in the Orthopedics and Traumatology Service (OTR) of the Centre Hospitalier Universitaire Vaudois (CHUV, Lausanne, Switzerland). General process parameters and technical specifications were compiled from the ad hoc investigational medicinal product dossier (IMPD) submitted to federal public health authorities in the context of the clinical trial of interest. The corresponding GMP manufacturing records were compiled in the Cell Production Center (CPC), the in-house accredited and authorized (i.e., since 2015) GMP manufacturing platform within the CHUV Service of Pharmacy. The corresponding HAC functional quality control records were compiled from the Unit of Regenerative Therapy (UTR) within the CHUV Plastic and Reconstructive Surgery Service (CPR). Appropriate data and information anonymization and data security protocols were used at all times during the study. 

#### 2.1.2. Ethical, Regulatory, and Clinical Protocols of the ACI CHUV Clinical Trial

The prospective and interventional clinical trial referenced hereabove had been approved by the local cantonal ethics committee (i.e., Vaud Cantonal Ethics Committee, CER-VD authorization No. 2015-00145). The clinical trial was registered following federal authorization by Swissmedic (i.e., Swissmedic authorization No. 2016TpP1005), the Swiss therapeutic products agency. The CHUV internal clinical trial reference was “Project ACI-OTR: Autologous cell therapy product: Human articular chondrocytes”. The patient inclusion criteria for participation in the clinical trial, as detailed in the ad hoc clinical protocols, are summarized hereafter:

- Patient age > 15 years and <50 years of age.
- Presence of symptomatic focal chondral and osteochondral defects of traumatic origin, grades III and IV of the defects according to ICRS classifications, and defect size < 15 cm^2^.
- The lesion may result from a failure of autologous osteochondral transplantation (i.e., mosaicplasty) or of microfractures.
- Presence of an adequate biomechanical environment (i.e., ligamentary stability, preserved or restored meniscus, neutral axial mechanical axis).
- Patient in good overall health, documented by an ASA score ≤ 2.
- Patient assessed as compliant and as capable of participating in pre/post-operative follow-up and reeducation.
- Patient consent for participation in the study.
- Procedure covered by basic health insurance or by accident insurance.
- Patient non-responsive to conservative treatment (>6 months).

The patient exclusion criteria for participation in the clinical trial, as detailed in the ad hoc clinical protocols, are summarized hereafter:

- All degenerative inflammatory pathologies and synovial pathologies (e.g., arthritis).
- Diffuse chondral lesions, of traumatic or non-traumatic nature (e.g., gonarthrotic).
- Non-favorable biomechanical environment (e.g., subtotal or total meniscectomy in the same compartment, ligamentary instability, deviation of the mechanical axis leading to an overload of the treated compartment).
- Qualified obesity of grade ≥ 2, with a body-mass index value > 35 kg/m^2^.
- Active tobacco product consumption habit.
- Consumption of hard recreational drugs.
- Bad compliance of the patient.
- Current participation in an alternative clinical trial.
- Compromised overall patient health, documented by an ASA score ≥ 3.
- Vulnerable populations.
- Active or planned pregnancy.
- Qualified allergy to porcine collagen, penicillin, or to gentamycin.
- Qualified seropositivity for HIV, HBV, HCV, or for *Treponema pallidum* (i.e., assessed by serological testing before biopsy harvest).
- Presence of growth cartilage (i.e., presence of an open epiphyseal growth plate) in adolescents 15–18 years of age.

The treatment of the included patients with the manufactured autologous HAC-based cell therapy products consisted of a two-stage surgical procedure eventually aiming to optimally favor the formation of new cartilage tissue, characterized by biomechanical properties close to those of healthy joint cartilage. Therefore, a biopsy of healthy cartilage tissue was firstly harvested in each patient from a non-weight-bearing area of the knee joint during an arthroscopic procedure, performed approximately 3.5 months prior to the implantation of the finished cell-based therapeutic product. The HACs were isolated from the tissue biopsy by a two-step enzymatic digestion reaction, were amplified by means of serial in vitro culture expansion, and were eventually formulated as an injectable viable cell suspension. Secondly, the autologous HAC suspension was implanted into the patient’s knee under arthrotomy, with the objective to favor the repair of the cartilage tissue damage by allowing the formation of functional and durable new cartilage tissue. The surgical procedure for cell therapy administration required the preparation of the injured area by debridement and the placement of a protective cover (i.e., an ad hoc biological membrane) over the lesion to hold the implant materials in place. The recommended cellular therapeutic product dose described by Brittberg et al. (i.e., 2 × 10^6^ cells/cm^2^ of lesion size or 66 µL of product/cm^2^ in the referenced clinical trial) was used, and the product implantation process was followed by an appropriate and individualized ad hoc patient rehabilitation program [22,35]. Briefly, the objectives of the clinical research were to evaluate the efficacy and the safety of HAC-based ACI for the treatment of focal chondral and osteochondral defects in the knee. The specific outcomes, measures, and the results related to the efficacy and the safety of the intervention studied in the referenced clinical trial shall be reported elsewhere upon completion of the clinical trial and analysis of the full patient follow-up results.

### 2.2. Original Data on Primary HAC Sourcing, Manufacturing, and Formulation

The summarized data presented in the first part of this study were mainly gathered from the available GMP records relative to the clinical lots of HAC-based cell therapy products, manufactured and administered in the CHUV in the context of the referenced clinical trial. The various steps of process validation (i.e., validation of the cell culture medium, cell culture conditions, HAC gain of function upon switching from two-dimensional to three-dimensional culture, cryopreservation) were not detailed herein, as they were already reported elsewhere for the most part [37]. 

#### 2.2.1. Biological Starting Material Procurement and Processing for In Vitro Cell Isolation

For the appropriate procurement of the healthy cartilage tissue biopsies from individual patients, tissue sampling and transport kits (i.e., biopsy kits) were provided to the qualified and experienced clinicians in the CHUV Orthopedics and Traumatology Service. The biopsy transport tubes contained 20 mL of sterile and conserved transport medium (i.e., DMEM medium, Sigma-Aldrich, St. Louis, MO, USA, supplemented with 1% *m/v* penicillin/gentamicin, Grünenthal, Aachen, Germany, and Hexal, Holzkirchen, Germany, respectively) and were delivered along with an ad hoc prescription form (i.e., CHUV form No. 70). Following the arthroscopic collection of the tissue biopsy (i.e., average cartilage tissue size of 4 × 12 mm) by the prescribing clinician, the isolated tissues were conditioned in the transport tubes and were immerged in the transport medium. The transport tubes were individually identified using standard institutional patient identity labels. For the eventual manufacture of the HAC-based finished products using autologous serum, blood samples were drawn from the patients at the time of anesthesia for the arthroscopy intervention and were conditioned in two ad hoc 5 mL glass tubes (BD, Franklin Lakes, NJ, USA). The tubes containing the blood samples were identified as described hereabove using patient labels and were packed along with the biopsy transport tubes. The collected biological materials and the corresponding documents were then transferred at ambient temperature under temperature monitoring from the operating suite to the in-house GMP manufacturing platform (CHUV CPC) in an isotherm transport container. 

Upon receipt of the biopsy kit in the CPC, following material liberation and entry into GMP production, the blood tubes were centrifuged at 3803× *g* for 10 min at ambient temperature, for recovery of the serum fraction. The serum was then aliquoted and stored at –20 °C until further use. Quality control retention samples of the isolated serum and of the biopsy transport medium were then processed for the investigation of microbiological quality (BD BACTEC TX™ 40, BD, USA). Following autologous serum batch clearance, specific GMP conformity certificates were established for these materials, to eventually be used as raw materials for finished cytotherapeutic product preparation. 

Parallelly, the cartilage tissue biopsies were transferred to sterile 10 cm diameter Petri dishes (BD, USA) and were rinsed twice using 10–15 mL of washing solution composed of phosphate-buffered saline (PBS) supplemented with 1% *m/v* penicillin/gentamicin (Grünenthal and Hexal, Germany). The aseptic processing of all biological samples took place in class A biocontainment modules (ISOCell Pro 1.8, Euroclone, Pero, Italy) located in class D GMP cell manufacturing suites. The cartilage tissue samples were then suspended in 0.2–1.0 mL of sterile DMEM medium (Sigma-Aldrich, USA) and were manually fragmented into small tissue particulates (i.e., <1 mm^3^) using a sterile scalpel (KLS Martins, Freiburg im Breisgau, Germany). The resulting tissue fragments were then transferred into a sterile cell culture T25 flask (25 cm^2^, Falcon^®^, Corning^®^, Glendale, AZ, USA) which contained 10 mL of pronase digestion solution (0.8 mg/mL, Roche, Basel, Switzerland) supplemented with 1% *m/v* penicillin/gentamicin (Grünenthal and Hexal, Germany). The T25 flasks were incubated at 37 °C for 1 h under gentle automatic shaking. Then, a volume of type II collagenase (i.e., appropriate to obtain a final concentration of 0.8 mg/mL collagenase, Invitrogen™, Thermo Fisher Scientific, Waltham, MA, USA) was added to the T25 tissue digestion flask, which was then further incubated at 37 °C overnight under gentle automatic shaking. 

Following the full incubation period, the complete digestion of the tissue samples was macroscopically confirmed by observation of the T25 tissue digestion flask. Then, the digestion solution containing the isolated cells in suspension was filtered on a 100 µm cell sieve (BD, USA) into a 50 mL sterile centrifugation tube (BD, USA) and was eventually aseptically transferred into a 15 mL sterile centrifugation tube (BD, USA). The resulting cell suspension was then centrifuged at 290× *g* for 10 min at ambient temperature. The resulting cell pellet was then washed twice using a PBS washing solution. The cells were then resuspended in 1–2 mL of complete cell culture medium for cell count determination on a Neubauer hemocytometer chamber. Relative cellular viability was determined using Trypan blue exclusion dye (Sigma-Aldrich, USA). The complete cell culture medium was composed of DMEM and HAM’s F12 (Sigma-Aldrich, USA) in 1:1 proportion, supplemented with 10% *v/v* human platelet lysate (hPL, Cook Regentec, Indianapolis, IN, USA), 2 mM L-glutamine (Sigma-Aldrich, USA), and 0.025 mg/mL L-ascorbic acid (Streuli Pharma, Uznach, Switzerland). The obtained cell pool was defined as the preliminary cell population at that time. Based on the preliminary cell population harvest cell counts, the cells were then seeded with 10 mL of complete cell culture medium, using a relative viable seeding density of 4–10 × 10^3^ cells/cm^2^, in a vented T25 cell culture flask (25 cm^2^, Falcon^®^, USA). The seeded cell culture vessels were then incubated in humidified incubators set at 37 °C under 10% *v/v* CO_2_. The first culture medium exchange procedure was performed on average 4 days after the cell seeding procedure and was further performed thrice weekly thereafter. The cell cultures were regularly macroscopically and microscopically monitored. The microscopic monitoring of the cell cultures was performed by operator observation under a phase contrast microscope, where the homogenous cell proliferation and the fibroblastic proliferative cellular morphology were iteratively confirmed. The endpoint cell culture harvest procedures were performed after the cell monolayers had reached 60–100% confluency levels. 

#### 2.2.2. Initial Cellular API Manufacturing Process Optimization and Validation Steps

In the context of the laboratory development of protocols to be transposed for the GMP manufacture of a cytotherapeutic product for cartilage repair promotion using in vitro monolayer cell expansion phases, two main aspects of the overall process generally require some optimization work. The first aspect consisted in the technical possibility of generating sufficient quantities of cellular bulk API materials to be used as raw materials for the preparation of the final product. The second aspect consisted in the obtention of cellular bulk API materials of an appropriate quality (i.e., viability, chondrogenic function) for human therapeutic use in cartilage regenerative medicine. The processes used for the preparation of the APIs (i.e., cell sourcing, cell manufacture, cell batch qualification) and of the finished products (i.e., formulation phase) as described in the context of the clinical trial mentioned herein were closely based on the works of Brittberg et al. [22,35]. 

However, various specifications were adapted, such as the use of commercial hPL instead of autologous patient serum as a cell culture medium supplement, which benefitted to both the HAC manufacturing yields and the chondrogenic cell function, as reported elsewhere [37]. Overall, multiple parameters of the API and of the finished product manufacturing processes were respectively and first optimized in the research laboratory in the UTR, before the technology transfer was performed toward the in-house CPC platform for GMP process validation. Key and critical aspects such as cellular morphological analysis, cell viability, cell count, and behavior of the cells during the in vitro monolayer expansion phases were investigated and served for preliminary process technical optimization and validation, using primary HAC cell types from 16 patients. Further functional investigation, technical optimization, and functional quality control validation related to the chondrogenic activity of the cells was performed, using HAC cell types from four patients. Acceptance criteria were established at that time and the processes were then transferred for GMP process validation to the CPC platform. Finally, the validation of the entire manufacturing process was performed by the CPC GMP platform, using primary HAC cell types from three patients, demonstrating the equivalence with the research laboratory manufacturing processes in terms of cellular viability, proliferative cellular morphology, cell proliferation behavior, and cellular chondrogenic activity increase upon switching from two-dimensional cultures to three-dimensional culture conditions. As previously mentioned, the results of the various steps of process validation were not detailed herein, as they were already reported elsewhere for the most part [37]. 

#### 2.2.3. Cellular API GMP Manufacturing and Controls for the CHUV ACI Clinical Trial

In order to generate sufficient quantities of clinical-grade cells for the constitution of appropriate cellular API lots, the cell populations isolated from the tissue biopsy and harvested following the first in vitro monolayer expansion phase were used for in vitro sub-cultures (Figure S1). For the first endpoint harvest procedure of the confluent cells, the spent cell culture medium was removed, and the cell monolayers were rinsed using 10 mL of sterile D-PBS (Invitrogen™, Thermo Fisher Scientific, USA). The rinsing medium was then removed, and the cells were enzymatically collected using 5 mL of 0.05% trypsin-EDTA (Invitrogen™, Thermo Fisher Scientific, USA) per vessel. The cell dissociation enzymatic reaction was favored by a 5 min incubation of the culture vessels at 37 °C, and the cell detachment was further stimulated by light manual stimulus of the culture vessels. Then, the enzymatic reaction was quenched with the addition of 5 mL of complete cell culture medium per vessel. The resulting harvesting cell suspension was collected for pooling in 50 mL sterile centrifuge tubes (BD, USA) and was then centrifuged at 290× *g* for 5 min at ambient temperature. The supernatant was then removed, and the cell pellet was resuspended in 5–10 mL of complete cell culture medium for cell count determination on a Neubauer hemocytometer chamber. Relative cellular viability was determined using Trypan blue exclusion dye (Sigma-Aldrich, USA), and an acceptance criterion of ≥90% cellular viability maintenance was applied. The obtained cell pool was defined as the cultured cell seed at that time. 

Using the cultured cell seed materials, an in vitro monolayer cell expansion step was then performed using a relative seeding density of 1.5–3.0 × 10^3^ cells/cm^2^ in a maximal amount of vented T75 cell culture flasks (75 cm^2^, Falcon^®^, USA). The cells were cultured in 15 mL of complete cell culture medium, which was exchanged thrice weekly. The cell culture vessels were incubated in humidified incubators set at 37 °C under 10% *v/v* CO_2_. The cell cultures were regularly macroscopically and microscopically monitored. The microscopic monitoring of the cell cultures was performed by operator observation under a phase contrast microscope, where the homogenous cell proliferation and the fibroblastic proliferative cellular morphology were iteratively confirmed. The cell culture endpoint harvest procedures were performed as previously described after the cell monolayers had reached 60–100% confluency levels. Total and viable cell counts were determined at that time. Then, the obtained cell pool was suspended in a cell cryopreservation solution (Biofreeze^®^ Biochrom, Bioswisstec, Schaffhausen, Switzerland, or CryoSOfree™, Sigma-Aldrich, Switzerland) and was conditioned in individual cryopreservation vials (Nunc™, Thermo Fisher Scientific, USA), with (1.0 ± 0.2) × 10^6^ cells/mL (i.e., 0.5–1.5 mL/vial) and with a minimum of two vials. The vials were then placed in constant-rate freezing devices (CoolCell™, Corning^®^, USA), which were themselves placed in a –80 °C ultralow temperature freezer for at least 4 h, to obtain a constant rate of cooling of –1 °C/min. Then, the frozen vials were transferred to the vapor phase of liquid nitrogen within temperature-monitored and level-monitored Dewar storage tanks assorted with an auto-filling supply of liquid nitrogen. The obtained cryopreserved material lot was defined as the master cell bank (MCB) at that time. 

The maximum number of in vitro passage procedures for the establishment of the MCB using the preliminary cell population was set at two. For the generation of appropriate quantities of cellular APIs, an additional in vitro monolayer subculture could be performed, using MCB materials and the same technical specifications described for the preparation of the MCB, for the establishment of a working cell bank (WCB). The maximum total number of in vitro passage procedures for the preparation of the bulk API lot using the preliminary cell population was set at four (Figure S1). The choice of this standardized limit was specifically based on preliminary results of in vitro cell type lifespan qualification (i.e., to guarantee the use of cells maintaining high proliferation capacities) while excluding the use of cell populations of relatively higher in vitro cell age, eventually prone to senescence (i.e., possibly due to genetic aberrations). Material specification sheets, certificates of analysis, and certificates of GMP compliance were prepared for each constituted cell bank lot.

During all of the open-container GMP manufacturing activities carried out in the class A modules, continuous module air pressure and particle count monitoring were automatically performed as in-process controls (IPC). Appropriate sedimentation and fingerprint boxes were iteratively used for microbiological post-process controls (PPC) throughout the manufacturing process. Appropriate liquid retention samples (i.e., cell culture medium and rinsing solutions) were iteratively segregated and conditioned for BACTEC™ microbiological post-process controls throughout the cell manufacturing process. Post-process controls were performed on the constituted cell bank lots, for the insurance of microbiological quality. An endotoxin detection test was performed (Endosafe^®^ PTS™/MCS™ Charles River, Wilmington, MA, USA) according to the European Pharmacopoeia (Ph. Eur.), and a limit value of <0.2 EU/mL was specified as an acceptance criterion. Mycoplasma detection assays were performed as post-process controls for specified pathogens (i.e., *M. hominis*, *M. pneumoniae*). Both the endotoxin and mycoplasma detection assays were performed on cell culture medium retention samples. Out-of-specification microbiological control results warranted specific investigations in the CHUV Microbiology Service (i.e., ISO 17025-accredited laboratory) and opening of a deviation. All of the GMP manufacturing data and related events were recorded in the appropriate batch records and in the batch files.

For each manufactured primary cell type, a post-process functional quality control was performed using a three-dimensional cell culture system and an analysis of chondrogenic gene expression levels, for confirmation of cellular API chondrogenic potential. For the preparation of the functional quality control assay materials, MCB vials were used for an ad hoc in vitro monolayer expansion phase in T75 cell culture flasks, as previously described for the cell banking steps. The obtained cell cultures were harvested, and the resulting cell suspensions were used to constitute stock cell suspensions in complete cell culture medium, with 5 × 10^5^ cells/mL. For three-dimensional cell culture system preparation, volumes of 1 mL of stock cell suspension were dispensed in conical-bottom 15 mL centrifuge tubes (Falcon^®^, USA), which were then centrifuged at 290× *g* for 10 min at ambient temperature to form cell pellets (i.e., one pellet/tube). The supernatant was removed, and the cell pellets were cultured as previously described for a maximum of 14 days in 2 mL of chondrogenic cell culture medium, which corresponded to complete cell culture medium supplemented with human transforming growth factor beta 1 (TGF-β1, at 10 ng/mL, Sigma-Aldrich, USA), insulin-transferrin-selenium (ITS, at 10 µg/mL, Sigma-Aldrich, USA), and dexamethasone (at 10^−7^ M, Sigma-Aldrich, USA). The chondrogenic cell culture medium was exchanged thrice weekly. At two defined timepoints of the chondrogenic culture phase (i.e., on the day of pellet constitution and after 16 days of culture, respectively), the pellets were harvested and were frozen at –20 °C in 0.5 mL of TRIzol, for subsequent RNA extraction and gene expression analysis. 

For the parallel extraction of RNA from the different samples (i.e., the different timepoints), the cell pellets were thawed at ambient temperature and were mechanically disrupted using a 1 mL syringe (BD, USA) mounted with an 18G needle (BD, USA). The resulting cell homogenate was submitted to lysis using TRIzol in the respective tubes. The RNA was precipitated using isopropanol (Sigma-Aldrich, USA), was then washed twice with 70% ethanol (Sigma-Aldrich, USA), and was then washed once with 100% ethanol. The RNA was then dried for 10 min at ambient temperature, recovered in distilled water (Millipore^®^, Merck, Darmstadt, Germany), and was quantified by spectrophotometry (NanoDrop™, Thermo Fisher Scientific, USA). Reverse transcription into cDNA was performed using 500 ng of RNA in a final volume of 50 µL, using 2.5 µM of hexamer randoms (Invitrogen™, Thermo Fisher Scientific, USA) and MultiScribe™ Reverse transcriptase 1.25 U/µL (Applied Biosystems, Thermo Fisher Scientific, USA), following the specifications and instructions of the manufacturer. The reverse transcription cycle conditions using a PCR Biometra T-personal (Biometra, Göttingen, Germany) were as follows: 25 °C for 10 min, 48 °C for 30 min, and 95 °C for 5 min. 

Real-time polymerase chain reaction (RT-PCR) was then performed in 96-well microplates (Greiner Bio One, Frickenhausen, Germany) on a StepOnePlus™ Real-time PCR System (Applied Biosystems, Thermo Fisher Scientific, USA). The reaction was performed using 1 µL of cDNA for a final volume of 20 µL, using the KAPA SYBR^®^ Fast (Kapa Biosystems, Roche, Switzerland), following the specifications and instructions of the manufacturers. Fluorescence was acquired using the following cycling conditions: 95 °C for 3 min (i.e., enzyme activation) and 40 amplification cycles (i.e., 95 °C for 3 s and annealing extension at 60 °C for 30 s). Each sample was run in triplicate, and the relative expression level for each gene was normalized to the GAPDH (i.e., coded by a housekeeping gene, used as an internal control) expression levels. The genes of interest for the evolutive analysis of gene expression levels during functional quality control assays were *Acan* and *COL2A1*. Gene expression levels were quantified using the 2^−ΔΔCt^ method, as described elsewhere [37].

#### 2.2.4. Cellular Finished Product Manufacturing and Controls

In order to manufacture the finished HAC-based therapeutic products in the form of a cell-laden injectable suspension, MCB materials were used for a final in vitro monolayer cell expansion phase. For each patient, the preparation of the finished cytotherapeutic product occurred at least 2 months after the initial arthroscopic procurement of the cartilage tissue biopsy. For the initiation of the cellular materials from liquid nitrogen cryogenic storage, the vials were rapidly placed for 2–3 min in a dry bath set at 37 °C. The vials were then entered in the class A module for processing and the cells were transferred into a 15 mL sterile centrifugation tube (BD, USA). The cell suspensions were diluted, were washed with D-PBS (Invitrogen™, USA), and were centrifuged at 290× *g* for 5 min at ambient temperature. The cells were then resuspended in 5 mL of complete cell culture medium for cell count determination on a Neubauer hemocytometer chamber, using Trypan blue exclusion dye (Sigma-Aldrich, USA) for relative cellular viability determination. Using the obtained cells, an in vitro monolayer cell expansion was then performed using a relative seeding density of (4.0–6.5) × 10^3^ cells/cm^2^ in a maximal amount of vented T75 cell culture flasks (75 cm^2^, TPP, Trasadingen, Switzerland). The cells were cultured in 15 mL of complete cell culture medium, which was exchanged thrice weekly. The cell culture vessels were then incubated in humidified incubators set at 37 °C under 10% *v/v* CO_2_. The cell cultures were regularly macroscopically and microscopically monitored. The microscopic monitoring of the cell cultures was performed by operator observation under a phase contrast microscope, where the homogenous cell proliferation and the fibroblastic proliferative cellular morphology were iteratively confirmed. The endpoint cell culture harvest procedures were performed as previously described, after the cell monolayers had reached 60–100% confluency levels.

For the endpoint harvest of the bulk cellular APIs, the spent cell culture medium was removed, and each culture vessel was rinsed with 10 mL of sterile D-PBS (Invitrogen™, USA). The cells were then enzymatically harvested with trypsin as described previously and were washed twice with 10 mL D-PBS. The cells were then resuspended in 5–10 mL D-PBS for cell count determination on a Neubauer hemocytometer chamber, using Trypan blue exclusion dye. A stock cell suspension was then prepared using the bulk cellular APIs and a solution of 0.9% NaCl (Bichsel, Switzerland), supplemented with 20% *v/v* autologous human serum, for the obtention of a final concentration of 3 × 10^4^ cells/µL. The formulated cell suspension was then directly conditioned in 1 mL Luer-Lok™ syringes (BD, USA) fitted with safety caps (B. Braun Medicinal, Melsungen, Germany). The unitary cell dose in the syringe was adapted to clinical needs as prescribed, namely depending on the size of the cartilage lesion to treat, using a relative dose of 2 × 10^6^ cells/cm^2^ of cartilage lesion. The finished cytotherapeutic product was then conditioned in a sealed sterile plastic bag, was appropriately labelled (i.e., identification of the product and of the patient), and was eventually transferred at ambient temperature under temperature monitoring from the CPC in-house GMP platform to the operating suite in an isotherm transport container.

Post-process controls were performed on the finished products or on liquid retention samples gathered during finished product preparation, for the insurance of the appropriate microbiological quality of the product. An endotoxin detection test was performed (Endosafe^®^), where a limit value of <0.2 EU/mL was specified. Mycoplasma detection assays were performed for specified pathogens (i.e., *M. hominis*, *M. pneumoniae*). Specification sheets, certificates of analysis, and certificates of GMP compliance were prepared for each constituted finished product lot. 

### 2.3. Establishment of Optimized and Parametric Technical Workflows for HAC-Based API and Finished Product GMP Manufacture

#### 2.3.1. Risk Analysis-Based Process Approach for Parametric Definition of the Process, including Controls and Criteria

Based on the contents of the ad hoc IMPD, which described the processes for primary cell sourcing, cell isolation, cell manufacture, and finished product manufacture, specific and general risk analysis matrices (RAM) were established. For the finished cytotherapeutic product, the general RAM established for HAC-based injectable products was adapted from the European Medicines Agency (EMA) Guideline EMEA/CHMP/410869/2006 “Guideline on human cell-based medicinal products”. Specific and general RAMs pertaining to API and to finished product processing further served for the renewed parametric definition of the considered manufacturing processes, with the inclusion of process controls and of specified acceptance criteria. Therein, critical process parameters (CPP) were defined as parameters exerting a critical effect on the quality of the final manufactured cell batch/product lot. Similarly, key process parameters (KPP) were defined as parameters exerting a key effect on the quality of the final manufactured cell batch/product lot. Eventually, key and critical quality attributes were established for the cellular API and for the finished cytotherapeutic product, respectively. 

#### 2.3.2. Synthetic Establishment of the Optimized Parametric Process Workflows

Based on the existing process workflows and on related controls, the established RAMs and process parameters were synthesized to establish the optimized parametric workflows covering the processes for primary cell sourcing, cell isolation, cell manufacture, and finished product manufacture (Figures S1–S5). Such processes were designed to cover all steps between the time of biopsy receipt in the CPC up to the shipping of the finished product to the operating theatre. 

### 2.4. Numerical Data Processing, Statistical Analysis of Data, and Data Presentation

All of the quantitative data from patient files and from GMP manufacturing batch records were reported as mean values assorted to corresponding standard deviations. Most of the quantitative data from the GMP batch records were graphically presented as box-and-whisker plots, wherein the box plots represented medians and quartiles, and the whiskers represented minima and maxima. For the statistical comparison of average values from two datasets, an unpaired Student’s t-test was applied, after the appropriate evaluation of the normal distribution of the data. A *p* value < 0.05 was retained as a base for the determination of statistical significance. The statistical calculations and/or data presentation were performed using Microsoft Excel, Microsoft PowerPoint (Microsoft Corporation, Redmond, WA, USA), and GraphPad Prism v. 8.0.2 (GraphPad Software, San Diego, CA, USA), respectively. 

## 3. Results

### 3.1. Results of Compilation and Data Analysis for Clinical Workflows, Patient Files, and GMP Manufacturing Records 

**Figure 1:**
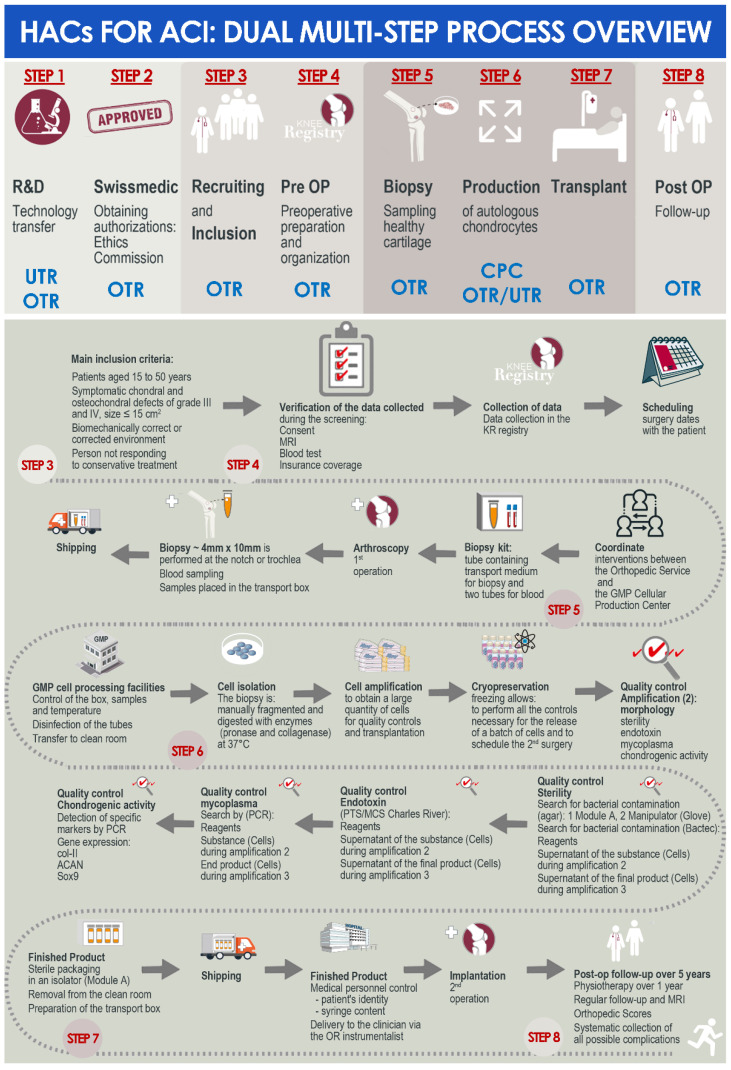
Schematic dual (i.e., general and detailed) multi-step process overview of the development and implementation steps for the considered HAC-based injectable cytotherapeutic products for ACI, within the context of the authorized clinical trial in the CHUV. ACI, autologous chondrocyte implantation; CHUV, centre hospitalier universitaire vaudois; CPC, cell production center; GMP, good manufacturing practices; HAC, human articular chondrocytes; MRI, magnetic resonance imaging; OTR, orthopedics and traumatology service; PCR, polymerase chain reaction; UTR, regenerative therapy unit.

A summarized overview of the available data related to the clinical workflow established for the referenced ACI clinical trial in the CHUV was prepared and is presented in Figure 1. Therein and despite the linear succession of events for biological material processing and cytotherapeutic care provision, critical importance was outlined for the effective multi-disciplinary collaboration and communication between the internal research and development, GMP manufacturing, and clinical orthopedic professional stakeholders within the institution (Figure 1).

A summarized overview of the available data related to patient demographic factors and articular cartilage lesion factors is presented in tabular form for the 47 patients included in the analysis (Table 1). Overall, 25 patients were included in the clinical trial due to chondral lesions, 22 patients were included due to osteochondral lesions, and the mean lesion planar size was of 4.7 ± 2.5 cm^2^ (Table 1).

**Figure 2:**
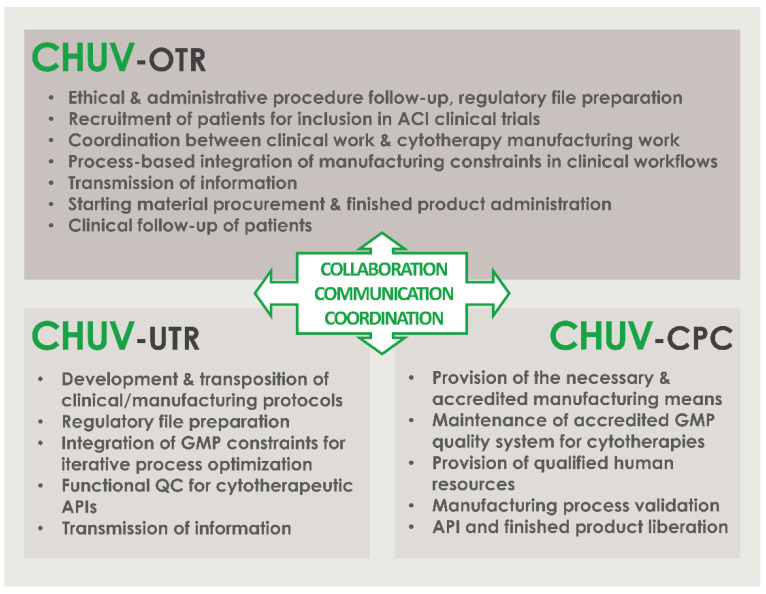
Simplified organigram specifying the roles and the responsibilities of the different CHUV professional stakeholders involved in the internal ACI clinical trial (i.e., research laboratory, GMP manufacturing platform, orthopedic clinical unit) within the CHUV. Critical importance is set on the effective collaboration, communication, and coordination between all of the stakeholders. ACI, autologous chondrocyte implantation; API, active pharmaceutical ingredient; CHUV, centre hospitalier universitaire vaudois; CPC, cell production center; GMP, good manufacturing practices; OTR, orthopedics and traumatology service; QC, quality control; UTR, regenerative therapy unit.

The patients included in the referenced clinical trial were regularly operated for cartilage tissue biopsy harvest between September 2017 and December 2021. We should note that operating rates were quantitatively negatively impacted by the COVID-19 pandemic in 2020 and in 2021, during which non-urgent surgeries were postponed in the CHUV. For an optimal comprehension of the roles and responsibilities of the different professional stakeholders involved in the internal ACI clinical trial (i.e., research laboratory, GMP manufacturing platform, orthopedic clinical unit), a simplified and annotated ad hoc CHUV organigram was prepared and is presented in Figure 2. 

**Figure 3:**
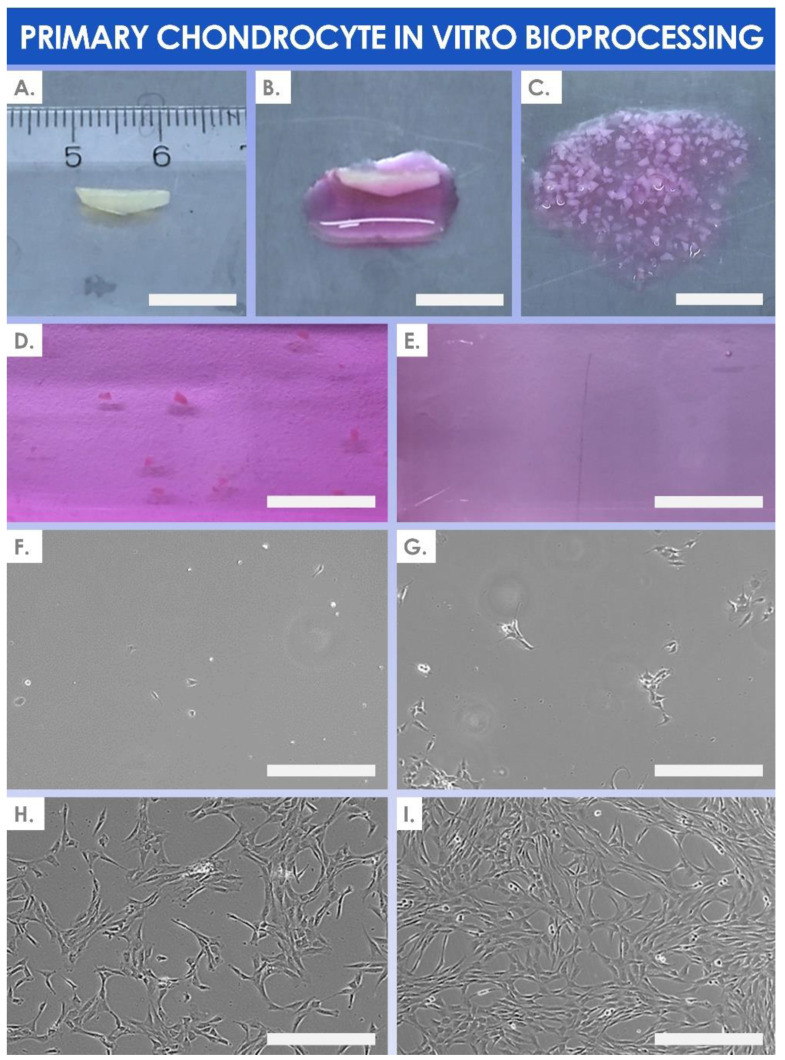
Photographic illustrative overview of the sequential mechanical and two-step enzymatic cartilage biopsy processing phases, followed by in vitro monolayer HAC expansion. (**A**) Procurement of the healthy cartilage tissue biopsy (i.e., size of 4 mm × 10 mm). Scale bar = 1 cm. (**B**) Humidification of the cartilage tissue biopsy for further processing. Scale bar = 1 cm. (**C**) Manual fragmentation of the cartilage tissue biopsy into < 1 mm^3^ fragments. Scale bar = 1 cm. (**D**) Two-step digestion of the cartilage tissue biopsy fragments with pronase and with collagenase. Scale bar = 1 cm. (**E**) Verification of complete cartilage tissue biopsy fragment digestion after overnight incubation with the lytic enzymes. Scale bar = 1 cm. (**F**–**I**) Photographic illustrative overview of sequential monitoring timepoints during the in vitro monolayer HAC culture expansion (i.e., cells at passage level 2, with photographs taken after 24 h, 2 days, 4 days, and 7 days of culture, respectively). Scale bars = 150 µm. h, hours; HAC, human articular chondrocytes.

As regards the original data related to in vitro cell isolation from the cartilage tissue biopsies, various elements from the available manufacturing records are presented hereafter. Notably, photographic records of tissue bioprocessing and of in vitro HAC cell culture are presented in the form of an illustrative overview in Figure 3.

**Figure 4:**
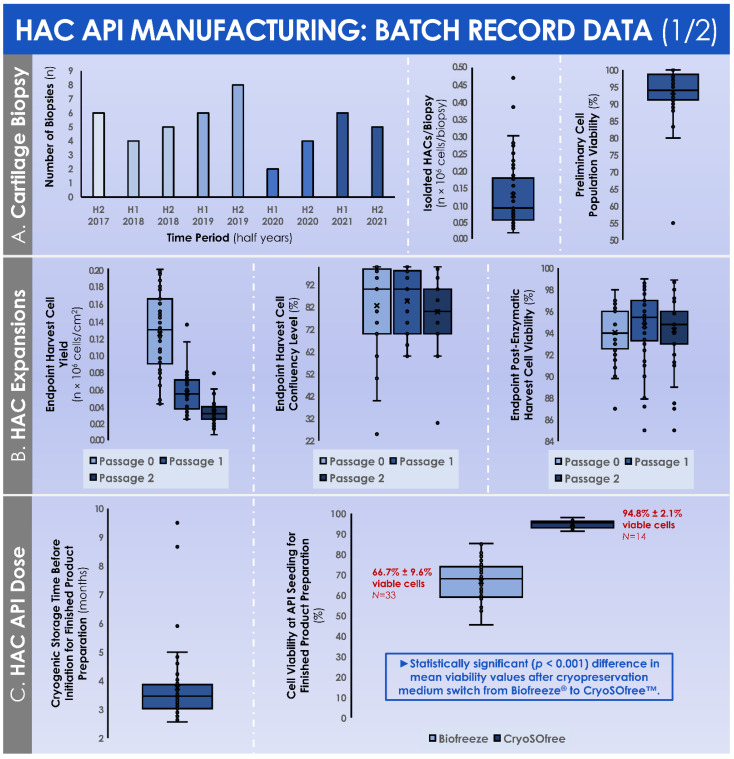
Original data from GMP manufacturing records relative to primary HAC (*n* = 47 cell types) isolation and manufacture for human investigational cytotherapeutic use. (**A**) Evolution of the numbers of biopsies performed between 2017 and 2021, quantitative data distribution for the obtained HAC cell counts after enzymatic biopsy processing, and quantitative data distribution for the obtained HAC relative cellular viability after biopsy processing for cell isolation. (**B**) Quantitative data distributions for the manufactured HACs relative to the endpoint harvested cell yields, the endpoint cell confluency levels, and the endpoint post-harvest relative cellular viability levels. (**C**) Quantitative data distributions for the manufactured HACs relative to the storage time-period between cell bank manufacture and finished product preparation, as well as the breakdown of the mean cell viability values at the time of initiation of HACs cryopreserved in Biofreeze^®^ medium or in CryoSOfree™ medium, respectively. A *p*-value < 0.05 was retained as a base for statistical significance determination. API, active pharmaceutical ingredient; GMP, good manufacturing practices; HAC, human articular chondrocytes.

The quantitative data relative to cartilage tissue biopsy processing and in vitro HAC manufacture, gathered from the GMP batch records, are summarily presented hereafter in graphical form. Following the mechanical tissue disruption and the two-step enzymatic processing of cartilage biopsies for primary in vitro HAC cell isolation, a mean number of (1.3 ± 1.0) × 10^5^ cells were isolated (Figure 4A). The enzymatically isolated cells in the preliminary cell populations were characterized by a mean relative viability fraction of 93.5% ± 7.5% viable cells at the time of quenching of the cell isolation reaction (Figure 4A).

**Figure 5:**
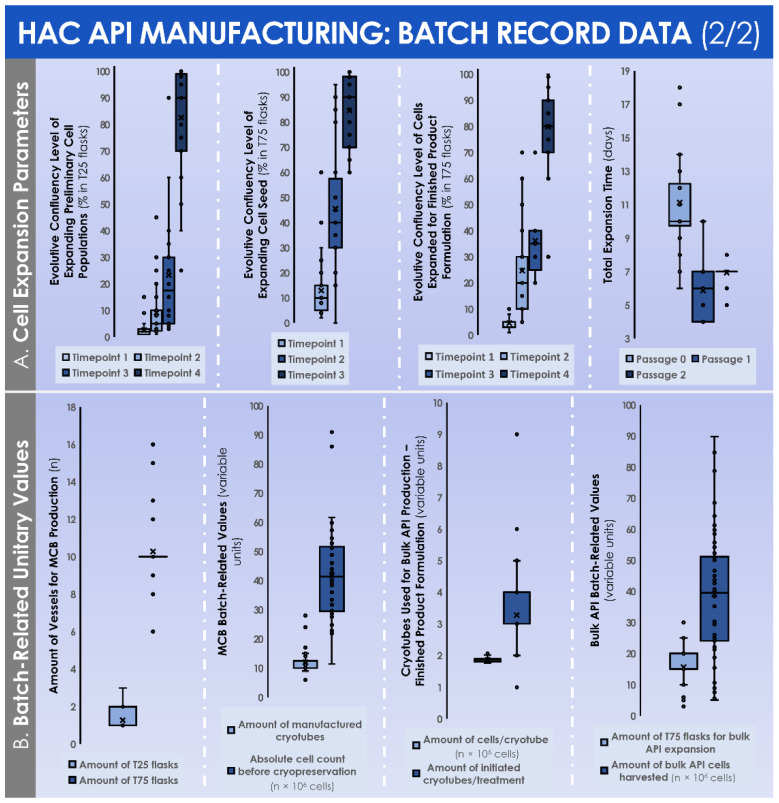
Original data from GMP manufacturing records relative to primary HAC (*n* = 47 cell types) isolation and manufacture for human investigational therapeutic use. (**A**) Quantitative data distributions for the manufactured HACs relative to the evolutive cell confluency level assessments at the various timepoints (i.e., defined as the microscopic observation of cultures during the medium exchanges) within each in vitro culture expansion phase, as well as the total culture time periods for each of the considered in vitro culture expansion phases. (**B**) Quantitative unitary data distributions for the manufactured HACs relative to the used materials and to the obtained harvest cell yields within MCB manufacture, as well as to the used materials and to the obtained harvest cell yields within finished product manufacture. API, active pharmaceutical ingredient; GMP, good manufacturing practices; HAC, human articular chondrocytes; MCB, master cell bank.

Over the course of the successive in vitro monolayer expansions of the HACs, the endpoint relative cell yields progressively diminished in value, attaining (1.2 ± 0.5) × 10^5^ cells/cm^2^ following the expansion of the preliminary cell population (i.e., P_0_ cells, expanded in T25 flasks), further attaining (0.6 ± 0.2) × 10^5^ cells/cm^2^ following the expansion of the cell seed (i.e., P_1_ cells, expanded in T75 flasks), and finally attaining (0.3 ± 0.1) × 10^5^ cells/cm^2^ following the expansion of the cells from the MCB (i.e., P_2_ cells, expanded in T75 flasks) (Figure 4B). Despite the relative and progressive reduction in endpoint post-expansion cell yields, the endpoint cell confluency levels remained stable throughout the passages (i.e., 82.6 ± 18.0%, 84.6 ± 14.3%, and 79.8 ± 14.7% for P_0_, P_1_, and P_2_ cell populations, respectively), with a similar evolution observed for endpoint relative cell viability after harvest (i.e., 94.1 ± 2.4%, 94.7 ± 3.3%, and 94.3 ± 3.0% for P_0_, P_1_, and P_2_ cell populations, respectively) (Figure 4B). During the monolayer HAC expansions, the evolutive confluency level assessments (i.e., various timepoints corresponding to the cell culture medium exchange procedures) produced quantitative values that followed the same trend throughout the considered in vitro passages (Figure 5A). 

The total culture time periods were found to be relatively superior for the expansion of the preliminary cell populations in the T25 flasks (i.e., 11.1 ± 3.0 days for P_0_ cells), as compared to both expansions in the T75 flasks (i.e., 5.9 ± 1.5 days for P_1_ cells and 7.0 ± 0.4 days for P_2_ cells) (Figure 5A). In order to perform the successive in vitro monolayer expansions of HACs, the mean numbers of used culture vessels were of 1.3 ± 0.5 T25 flasks to expand the preliminary cell population (i.e., P_0_ cells), of 10.3 ± 1.7 T75 flasks to expand the cell seed (i.e., P_1_ cells), and of 15.6 ± 6.0 T75 flasks to expand the cells from the MCB (i.e., P_2_ cells) (Figure 5B). 

**Figure 6:**
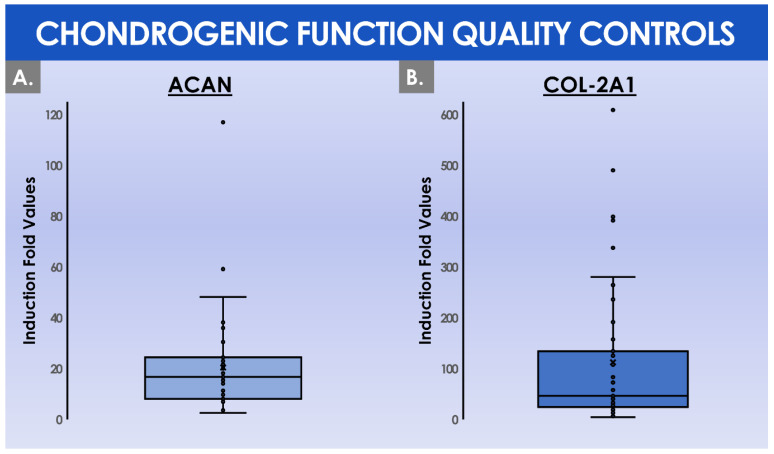
Comparative quantitative results of functional parameter (i.e., chondrogenic gene evolutive expression levels) QC assays for the cellular APIs, outlining inter-patient variability. (**A**) Induction fold values for the *Acan* gene expression upregulation (i.e., using the ΔΔCT method) between day 1 (i.e., baseline) and day 16 (i.e., endpoint) of three-dimensional cell cultures in chemically induced chondrogenic conditions. (**B**) Induction fold values for the *COL2A1* gene expression upregulation between day 1 and day 16 of three-dimensional cell cultures in chemically induced chondrogenic conditions. The quantitative data from the functional QC assay were presented for 42 patients. Several patients were excluded from the analysis due to a change in the chondrogenic medium composition. API, active pharmaceutical ingredient; QC, quality control.

During the constitution of the MCB lots, the mean total cell count within the harvested cell pools before MCB cryopreservation was of (41.8 ± 15.6) × 10^6^ cells (Figure 5B). These cells were then used to constitute MCB lots of 11.9 ± 4.2 cryotubes/lot, with (1.9 ± 0.1) × 10^6^ cells/cryotube (Figure 5B). On average, the manufactured MCB lots were cryogenically stored for 3.8 ± 1.3 months before the initiation for the manufacture of the finished product (Figure 4C). Then, the average number of MCB cryotubes initiated for finished product manufacture was of 3.3 ± 1.3 cryotubes/lot (Figure 5B). We should note that the cell viability levels upon initiation were statistically different (*p* < 0.001) depending on the used cryopreservation medium (i.e., the medium switch was performed from Biofreeze^®^ to CryoSOfree™ after the 33^rd^ patient due to a supply chain discontinuation, Figure 4C). Following the final in vitro HAC monolayer expansion in T75 flasks for the preparation of the bulk cellular API, the average total cell count within the harvested cell pools was of (42.2 ± 17.8) × 10^6^ cells (Figure 5B). Finally, the mean finished product individual quantity delivered to the operating room was of 0.88 ± 0.45 mL/patient. As regards the results of the chondrogenic gene expression induction, assessed during the functional quality control step of the manufactured HAC primary cell types, important inter-patient variability was evidenced (Figure 6).

Specifically, the mean induction fold values of the chondrogenic genes *Acan* and *COL2A1* were determined to reach 20.5 ± 20.0 and 112.5 ± 143.7, respectively, when comparing the expression levels at day 0 and day 16 of the three-dimensional cell culture, respectively (Figure 6). Considering the overall process of GMP manufacture, a certain number of deviations were recorded in the ad hoc batch records. A summarized overview of the recorded deviations and of the related corrective actions is presented in tabular form in Table 2.

Of high importance for the overall success of cytotherapeutic care provision, it should be noted that despite the recording of various deviations in the cellular API and in the cytotherapeutic finished product manufacturing processes, no cases of failure to deliver a liberated finished product lot were recorded (Table 2).

### 3.2. Results of the Standardized, Risk Analysis-Based, and Parametric Process Definition

In order to better approach the considered GMP processes for API and for finished product manufacture and processing, general and detailed process workflows relative to the referenced clinical trial were presented in Figure 1. For clarity of the used nomenclature and of definitions, a summarized technical workflow detailing the correspondence between in vitro cell passage levels, cell bank tiers, and material-related nomenclature was established and was presented in Figure S1. In order to subsequently subdivide the processes for API and for finished product manufacture into distinct steps, illustrated technical workflows presenting the sequential steps of the applied processes, as described in the ad hoc IMPD, were established and presented in Figures S2–S5. 

Then, following the establishment and the careful consideration of the graphical materials described hereabove, general and specific risk analyses were performed for the various steps of the considered processes and were presented in the form of risk analysis matrices (RAMs). In detail, a general RAM was established for the assessment of the sourcing, procurement, and in vitro culture initiation of primary HAC cell types (Table S2). A general RAM was also established for the assessment of the banking of primary HAC cell types for cellular API manufacture (Table S3). A specific RAM was further established for the assessment of the microbiological safety (i.e., excluding viruses) of primary HAC cell types, considering the cells as cryopreserved APIs for medicinal products (Table S4). Finally, a general RAM was established for the assessment of HAC-based injectable products for human ACI use, as adapted from applicable EMA guidelines (Table S5). 

**Figure 7:**
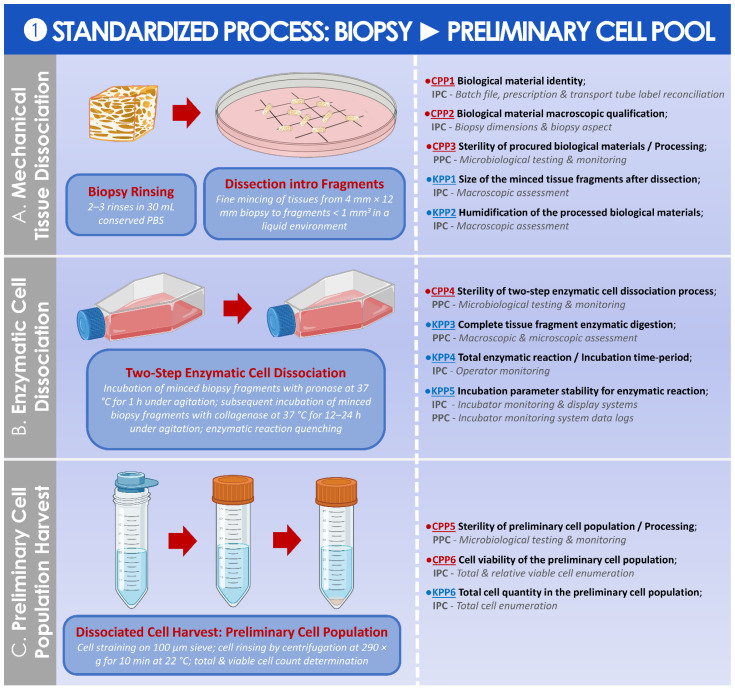
Standardized parametric and controlled process overview for the obtention of the preliminary HAC cell population from the cartilage tissue biopsy. (**A**) Mechanical cartilage tissue dissociation process, in preparation for the enzymatic tissue treatment. (**B**) Two-step enzymatic cartilage tissue treatment for in vitro HAC cell dissociation. (**C**) In vitro isolation of the preliminary HAC cell population. The established CPPs, KPPs, IPCs, and PPCs are further defined in the supplementary document–Process Parameters: Table SPP1. API, active pharmaceutical ingredient; CPP, critical process parameter; HAC, human articular chondrocytes; IPC, in-process control; KPP, key process parameter; PBS, phosphate-buffered saline; PPC, post-process control.

The final step of this study was to present the standardized (i.e., as currently implemented in the CHUV) or some optimized (i.e., propositions for potential further implementation) parametric and controlled processes in the form of illustrated step-wise process elements, after close consideration of the existing elements of the GMP process and of the newly established RAMs. These parametric processes allowed for a breakdown of the various steps, the identification, and the assessment of the importance of individual parameters (i.e., key or critical influence of a given parameter on the quality of the manufactured materials). Importantly, the technical specifications and the selected parameters presented within the standardized or the optimized parametric processes were all based on the summarized analyses of GMP record data presented in the first part of this study. Therefore, the adequacy and the robustness of the presented parametric processes may be considered with a relatively high degree of confidence, given the fact that they were based on the study of 47 separate HAC cell types, manufactured by a defined GMP platform and an ad hoc system. In particular and due to the real-scale nature of the GMP data used herein as a working basis, the presented processes may be considered as relatively and qualitatively superior to theoretical parametric processes or to processes established and validated on a small number of biological samples before implementation. Overall, the multi-disciplinary experience and knowledge gained in the context of the referenced ACI clinical trial were leveraged in this study, tentatively providing a technical basis (i.e., outlining key and critical process points) for researchers and clinicians considering or endeavoring a similar implementation of autologous HAC manufacture for therapeutic ACI in public hospitals. In detail, a standardized parametric process was established and is presented for the preparation of the preliminary cell pool from the harvested cartilage tissue biopsy (Figure 7). 

**Figure 8:**
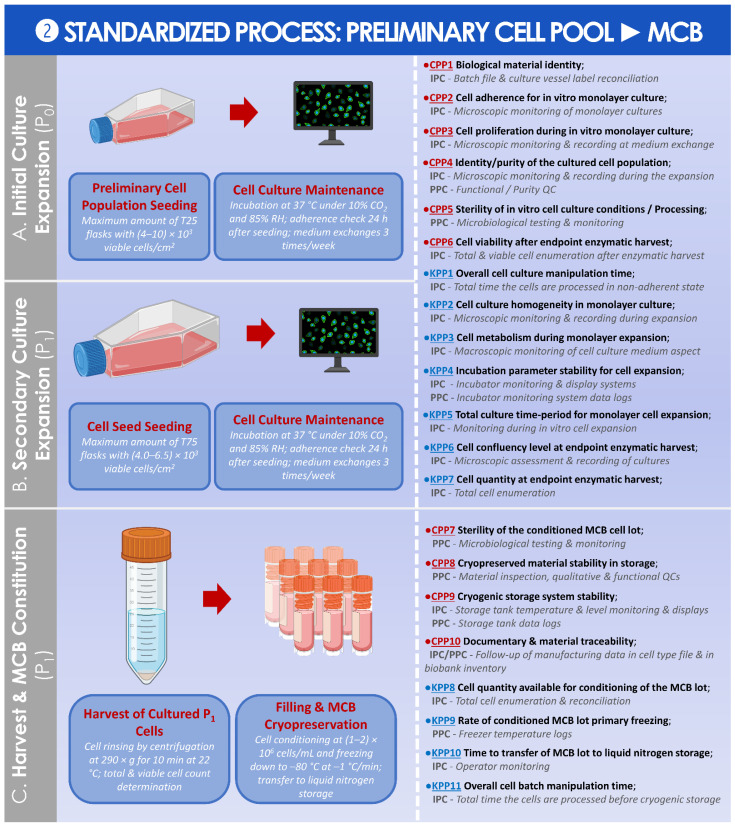
Standardized parametric and controlled process overview for the obtention of the MCB from the preliminary HAC cell population. (**A**) Initial in vitro culture expansion of the preliminary HAC cell population for the obtention of the cell seed. (**B**) Secondary in vitro culture expansion of the cell seed for the obtention of the cells used in MCB batch constitution. (**C**) Harvest and cryopreservation of the obtained cells for the establishment of the MCB. The established CPPs, KPPs, IPCs, and PPCs are further defined in the supplementary document–Process Parameters: Table SPP2. API, active pharmaceutical ingredient; CPP, critical process parameter; HAC, human articular chondrocytes; IPC, in-process control; KPP, key process parameter; MCB, master cell bank; PPC, post-process control; QC, quality control; RH, relative humidity.

A standardized parametric process was then established and is presented for the preparation of the MCB from the preliminary cell pool (Figure 8). 

**Figure 9:**
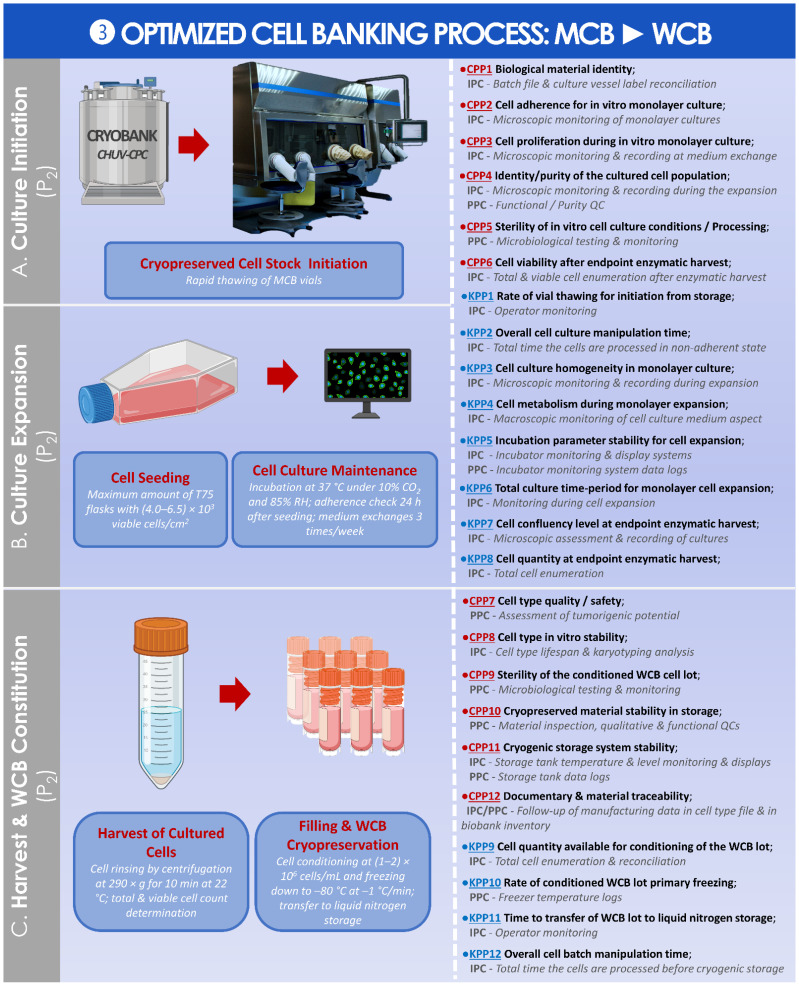
Optimized parametric and controlled process overview for the obtention of the WCB from the MCB. (**A**) Culture initiation of MCB materials for in vitro cell expansion. © CHUV-CPC. (**B**) Single in vitro cell expansion. (**C**) Harvest and cryopreservation of the obtained cells for establishment of the WCB. The established CPPs, KPPs, IPCs, and PPCs are further defined in the supplementary document–Process Parameters: Table SPP3. API, active pharmaceutical ingredient; CHUV, centre hospitalier universitaire vaudois; CPC, cell production center; CPP, critical process parameter; HAC, human articular chondrocytes; IPC, in-process control; KPP, key process parameter; MCB, master cell bank; PPC, post-process control; QC, quality control; RH, relative humidity; WCB, working cell bank.

An optimized parametric process was then established and is presented for the preparation of the WCB from the MCB (Figure 9).

**Figure 10:**
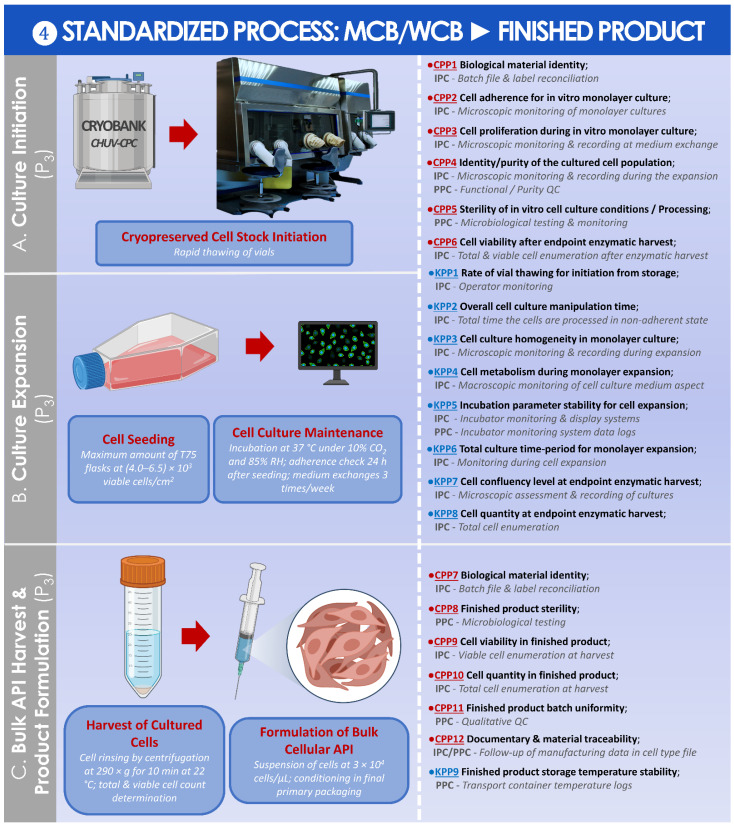
Standardized parametric and controlled process overview for the obtention of the finished product from the MCB or the WCB. (**A**) Culture initiation of MCB/WCB materials for the final in vitro cell expansion. © CHUV-CPC. (**B**) Single in vitro cell expansion. (**C**) Harvest and formulation of the obtained cells for the obtention of the finished product. The established CPPs, KPPs, IPCs, and PPCs are further defined in the supplementary document–Process Parameters: Table SPP4. API, active pharmaceutical ingredient; CHUV, centre hospitalier universitaire vaudois; CPC, cell production center; CPP, critical process parameter; HAC, human articular chondrocytes; IPC, in-process control; KPP, key process parameter; MCB, master cell bank; PPC, post-process control; QC, quality control; RH, relative humidity; WCB, working cell bank.

A standardized parametric process was finally established and is presented for the preparation of the finished HAC-based injectable product from the MCB or from the WCB (Figure 10). 

For each parametric process segment, the established process parameters, controls, targets, methods, and acceptance criteria were detailed in Tables SPP1–SPP4 in the supplementary document “Process Parameters”. Finally, key and critical quality attributes were determined for the cryopreserved form of the HAC APIs and for the finished HAC-based therapeutic product, respectively, and were presented in Tables SQA1 and SQA2 in the supplementary document “Quality Attributes”.

## 4. Discussion

### 4.1. Critical Importance of Interdisciplinary Collaboration, Communication, and Coordination of Professional Stakeholders for the Successful Clinical Implementation of HAC-Based ATMPs/ATIMPs 

As previously mentioned, the highly encouraging preliminary clinical results and the full technical success of autologous HAC-based cytotherapeutic care provision within the context of the referenced clinical trial in the CHUV have enabled a robust assessment of the overall process quality. Specifically, despite the selected technical margins of potential optimization identified in the general multi-step process (i.e., as they exist in all GMP processes), all 47 prescriptions of HAC-based therapeutic products have, in fine, been followed by the delivery of liberated finished products meeting all predefined safety and quality requirements (Figure 1). The fact that no instance of repeat cartilage biopsy harvest was necessary for the included 47 patients constituted an overall critical marker of process adequacy and effectiveness, which is sufficient in the context of university hospital investigational ATMP (ATIMP) manufacture for in-house clinical application.

The founding principles of such technical success in the context of investigator-initiated clinical trials may be firstly identified around the clear definition of the roles and the responsibilities of the different professional stakeholders involved in the clinical research, with the provided example of a simplified intra-institution organigram (Figure 2). Therein, the effective multi-disciplinary collaboration, communication, and coordination between the internal research and development, GMP manufacturing, and clinical orthopedic professionals guarantee the required continuity in the high-quality cytotherapeutic care provision (Figure 2). To further detail the parameters and the prerequisites of the adequate operational function of such supra-service collaborative activities, a short and structured list of fundamentals is provided hereafter. Therefore, an adaptation of the 7C concept, reported by Iancu and Kandalaft in the context of establishing a GMP cell therapy platform in a hospital setting, was established and proposed for the guaranty of the optimal provision of cytotherapeutic care within such ACI clinical trials [16]. The various points, derived from the aggregated experience of the three stakeholders described in Figure 2, are as follows:

- Communicate; establish clear, precise, and traceable transmission of information and data between all units for the appropriate meeting of general and specific clinical needs; establish regular exchanges for iterative assessments and optimization of process quality.
- Compliance; regularly assess the continued availability of accredited manufacturing means, the continued compliance of all activities with applicable institutional/legal frameworks and clinical trial authorizations, and the continued monitoring of ethical compliance with defined protocols.
- Clarify; establish clear roles and responsibilities of the involved personnel and units; identify individual responsibilities at each step of the considered processes.
- Collaborate; mutualize resources for an enhanced detection of risks and provision of efficient solutions; collaboration of research and clinical units for understanding of clinical needs and provision through development of adequate solutions; collaboration of research and GMP manufacturing units for transposition of the developed processes in response to clinical needs; collaboration of GMP manufacturing and clinical units for meeting of individual patient needs and clinician requirements.
- Coordinate; coordinate activities between GMP manufacturing and clinical units for meeting of clinician expectations and patient needs; continually seek to identify potential process gaps to be corrected by complementary responsibility attribution.
- Control; iterative and step-wise verification of information comprehension following communication between the stakeholders; verification of resulting action performance.
- Check; validation and revalidation of the processes after technical specification updates or material changes; regular reassessment of the entire process for verification of the adequation between objectives and available data/records/results.

Repartition of the various presented points of the adapted 7C list between the stakeholders (i.e., by means of a responsibility matrix) would be considered as inappropriate in the present case, as each and every stakeholder must take part in all individual activities. Taken together, these elements are useful and necessary for the integrative collaboration of all the involved multidisciplinary specialists, in order to ensure the optimal quality and excellence in the provided cytotherapeutic care. 

### 4.2. High Inter-Patient Variability: Standardized Manufacturing Processes for Patient-Specific Cytotherapies 

Although the analyzed GMP manufacturing processes are standardized and the finished therapeutic products are defined as standardized transplant products in Swiss reference texts, the inter-patient variability outlined in the presented data suggests that finished products should be considered as patient-specific transplants, rather than a “standardized” product. Indeed, highly variable total HAC cell counts were recorded at the time of the preliminary cell population enzymatic isolation from the cartilage tissue biopsies (Figure 4A). Furthermore, high HAC functional variability was outlined as regards chondrogenic gene expression induction within functional QC assays (Figure 6). Such results may be attributed in part to the inter-patient variability normally present in a human population selection, as well as to the specific status of cartilage tissues in each patient, which was in most cases relatively compromised for various diagnosis-related reasons. Therefore, the specific definition of precise quantitative acceptance criteria for functional QCs cannot tangibly be performed at this stage and in this setting, based on the variability observed in evolutive chondrogenic gene expression levels in vitro (Figure 6). This point has served as a basis for the specification of a positive difference in the evolutive chondrogenic gene expression levels within the timeline of the described functional QC assay, but without a determined and quantitative threshold of acceptance.

Specifically, the choice of the two genes *Acan* and *Col-2A1* was guided by the fact that the respectively related proteins are main constituents of hyaline cartilage, which should be part of the repaired or restored tissue in case of effective healing. These genes are among the most cited in the ad hoc literature, along with those related to collagen 1, which could potentially be further included in analyses for the assessment of cartilage quality. Overall, the assessment of chondrogenic gene expression in an in vitro three-dimensional cell culture assay in this study served the purpose of verifying that the cellular materials were able to revert to matrix-producing activities, which were transiently diminished during the in vitro monolayer expansion steps. However, preliminary internal results have indicated that no direct correlation could be evidenced between the gene expression levels in the presented functional QC assays and the clinical efficacy outcomes (i.e., with cartilage imagery assessments during patient follow-up after three years, results not shown). Therefore, it may be currently stated that the presented functional QC assay by genetic expression assessment may serve as an initial basis for a potency assay, but that much more development work is required for this objective, as the gene expression level in vitro is currently not predictive of clinical functional/therapeutic effects in the presented settings. Indeed, it is arduous to compare the behaviors of the produced HACs in vitro (i.e., short timeframe of culture, chemical stimulation of the cells) and in vivo after clinical implantation (i.e., patient follow-up period over several years, biomechanical stimulation of the cells, high cell density, support membrane colonization) in a standardized assay aiming to determine potency. Therefore, experimental setups using mechanobiological and histological endpoints for example appear attractive as alternative functional assays potentially comprising a clinically predictive component, yet the routine GMP manufacture of HACs requires the implementation of simple, robust, and efficient methods and models. 

Both of the readouts mentioned hereabove to describe the inter-patient variability (i.e., cell count of the preliminary cell population and functional QC) may be considered as primarily dependent on intrinsic characteristics of the individual primary HAC cell types, and secondarily dependent on the in vitro manufacturing processes. Indeed, process-related high variability was notably outlined in the analysis of data on cell viability upon final initiation (i.e., with a clear dependence on the type of cryopreservation medium) and the total amount of harvested bulk cellular API (Figure 4C and Figure 5B). As previously mentioned, none of these aspects have led to a failure to deliver the prescribed finished product to the operating theatre, yet technical margins of amelioration remain as regards process robustness in specific steps, as in all GMP manufacturing processes. Notably, the use of multi-tiered cell banking of HACs (i.e., in MCBs and WCBs) would constitute a valid approach to guarantee the endpoint obtention of sufficient amounts of cells for finished product preparation, provided that the in vitro lifespan of the HAC cell type allows it (Figure 5B). Furthermore, based on the available in-house experience around routine processing of primary cell cultures and standardization thereof, high interest is currently set on the qualification of an automated cell enumeration solution (e.g., ADAM™-MC, Countess™ 3, or NucleoCounter^®^ NC-200 equipment) to replace the existing manual operator cell enumeration steps. Indeed, such solutions would enable the generation of GMP-compatible reports on cellular viability and total cell count, as well as the suppression of the key inter-operator variability or bias which classically characterize manual hemocytometer cell enumeration.

Overall, consideration of the multiple variability sources (i.e., patients, operators, manufacturing process) prompts the careful assessment of the overall approach of HAC-based therapeutic product preparation for ACI. As for similar autologous cytotherapeutic applications, specific care should be taken at the time of process definition to avoid a rigid definition of targets and of acceptance criteria, as they exist in classical small therapeutic drug manufacturing processes. This in turn allows for the maintenance of high autologous cytotherapeutic product safety and quality levels, while at the same time allowing for the liberation of biological materials which may be considered as outliers in terms of individual process parameters and for the minimization of the amounts of deviations. Therefore, while process parameters and targets may be very precisely defined, acceptance criteria should always be specified as narrow as possible, but as wide as necessary, based on the analysis of the available manufacturing data and records as presented herein. 

### 4.3. Confirmation That the Use of hPL Is Appropriate for Primary HAC Culture in Clinical ACI Applications

As previously mentioned, the implementation of hPL as a cell culture medium supplement for HACs was performed for technical, regulatory, and quality reasons within the necessary manufacturing activities in view of ACI within the referenced clinical trial in the CHUV [37]. From a technical viewpoint, a comparative evaluation of fetal bovine serum (FBS), hPL, and aHS as culture medium supplements had been previously performed in-house on primary HAC types (*n* = 16), demonstrating the equivalence or the superiority of hPL to FBS in terms of HAC manufacturing yields within the in vitro passage levels of interest and in terms of functionality restoration in three-dimensional culture (i.e., histological and genetic expression readouts) [37]. Additionally, numerous recent investigations have provided strong evidence to the quality and the applicability of hPL as a chondrocyte in vitro culture medium supplement, in view of eventually phasing out animal-based materials [38,39,40,41,42,43,44,45,46,47,48]. Therefore, based on the addition of all these elements, the composition of Brittberg’s original in vitro cell culture medium was adapted for the clinical trial referenced herein, with the use of 10% *v/v* commercial hPL instead of aHS [22,37]. Furthermore, such adaptations may be justified in light of the efforts made to substitute animal-derived raw and ancillary materials in the manufacturing processes of biological therapeutic products. Therefore, the removal of FBS from the process already allows to lower the risk level relative to contamination of materials with viruses of bovine origin for example. A similar consideration was taken into account at the time of the switch from a DMSO-based cell cryopreservation medium to the defined mediums described herein. During the study, a supply chain discontinuation occurred for the cryopreservation solution retained and validated for the storage of HACs (i.e., Biofreeze^®^ medium). However, this occasion provided the grounds for the qualification, the validation, and the integration of the CryoSOfree™ medium into the HAC manufacturing process, which yielded a clearly positive impact on the post-initiation cell viability levels (Figure 4C). By extension, additional optimization measures could further be taken for the replacement of porcine trypsin with a defined cell dissociation reagent such as TrypLE™ or Accutase^®^, following the appropriate material qualification and comparative validation.

Two caveats are however specified hereafter concerning the use of hPL, pertaining to direct manufacturing costs and to the overall quality of the used materials. Indeed, commercial hPL products have suffered from drastic price increases in the past years, reportedly driven by iterative updates in the ad hoc purification and sterilization processes for GMP-compatible products. As the direct manufacturing costs relative to cell culture medium preparation already represent an important portion of the aggregated finished cytotherapeutic product manufacturing costs, it is foreseeable that the rise of hPL acquisition costs will further drive the augmentation of the overall cost of the manufacturing process. Secondly, with regard to the overall quality of the supplied hPL products, alternative considerations to standard criteria of clinical-grade or GMP compliance may be taken into account, such as the influence of donor nutritional habits, hormone intake, or therapeutic and recreational drug use on the final composition of the hPL batch. Therefore, the use of allogeneic pooled hPL as an ancillary manufacturing material comes with similar risks for the patient as the receipt of a blood transfusion (i.e., quite safe, but not attaining 100% safety) and could potentially be mentioned within the patient information documentation, where applicable. Such aspects, although not classified as key or critical with regard to the general quality of the cell culture supplement, may bare mid-term or long-term impacts on the sustainability of sourcing for such materials. Such aspects should be kept in mind at the time of switching from animal-based to human-based materials, and defined synthetic alternatives should be preferred, provided that these are qualified and available. 

### 4.4. Possibility to Optimize HAC Cell Banking Strategies for Enhanced Material Sustainability: Multi-Tiered Primary Cell Banking

When considering cartilage regenerative medicine and the wide variety of cellular chondrogenic APIs investigated within (i.e., adult chondrocytes, stem cells, embryonic or fetal progenitor cells), the sustainability aspect of therapeutic biological material sources (i.e., autologous or allogeneic) is always a central technical focus point [49,50,51,52,53,54,55,56,57]. Based notably on the average results of MCB lot size reported in the first part of this study, the use of a single cryopreserved cell bank tier is assessed as sufficient in most cases (Figure 5B). Indeed, in the case of a repeated need for therapeutic HACs by a patient (i.e., subsequent cartilage lesion or need for corrective surgery of the primary lesion treatment), sufficient amounts of MCB vials would remain for the preparation of one or two additional finished product doses. However, in the case of a particularly low HAC manufacturing yield during the MCB lot establishment (i.e., due to inter-individual variability), the sub-tiering of the biological material stocks into a WCB would be necessary, even for the initial preparation of a first finished product lot. In this case, multi-tiered HAC cell banking would not be a technical option as proposed in the optimized and parametric processes established herein, but an immediate technical necessity (Figure 9). Based on the in vitro HAC lifespan validation results obtained during hPL qualification phases, it has been determined that an additional in vitro cell passage procedure (i.e., to constitute a WCB) is technically possible (results not shown) [37]. 

Bearing in mind the rapid ageing of the general population and the increase in the prevalence of cartilage-related affections, a long-term strategy could be devised where all the isolated HAC cell types would be serially expanded to constitute MCBs and WCBs. This approach would prove highly sustainable and would contribute to minimize the number of biopsy harvesting operations undergone by the patient, since the biopsy would be performed only once. However, despite the reasonable direct costs entailed by systematic multi-tiered HAC banking, the overall costs of cryogenic storage and the effective material usage would need to be factored in. In all probability, the economic constraints would not allow for such an approach to be adopted in a public hospital setting, but could be considered in a for-profit clinical setup, for the on-demand provision of autologous cytotherapeutic products. 

### 4.5. Advantages of Risk Analysis-Based and Quality-Oriented GMP Manufacturing Approaches for HAC-Based Therapeutic Products in a Public Hospital Setting

Current clinical applications of cell-based therapeutic products in hospital settings may be performed under a variety of programs, regulatory classifications, authorizations or exemptions, yet quality-oriented focus imposes the compliance of the biological product manufacturer with current GMPs (cGMPs) [58]. While specific legal bases and reference documents have been established in the European Union for the GMP manufacture of ATMPs, applicable Swiss texts generally consist in the federal laws on therapeutic products and on transplantation, respectively, with references made to EU framework documents [59,60,61,62,63,64,65,66]. Although specific parameters and criteria are adopted depending on the processed materials and on the considered therapeutic applications, shared technical bases with regard to the insurance of safety and quality of the liberated products exist for all GMP-compliant platforms. This aspect is illustrated in-house in the CHUV CPC, a high-efficiency multi-product manufacturing platform, which has routinely manufactured (i.e., following medical prescription) autologous chondrocyte-based suspensions for ACI, autologous platelet-rich plasma (PRP) for tendinopathies and arthropathies, as well as simple or composed autologous cellular skin substitutes and allogeneic progenitor cell-based early wound coverage solutions for burns and wounds [58]. 

In addition to the meeting of basic regulatory requirements, the advantages of adopting risk analysis-based and quality-oriented GMP manufacturing approaches in public hospital settings comprise the ease of transposability of the considered process in an industrial setting. Specifically, while the contributions of public hospitals to the discovery and the primary development of innovative cell-based therapies have been historically recognized, it is often not part of the mission of a public institution to develop and to market finished therapeutic products. In such cases, private partners need to be identified and qualified for the continued development of products (i.e., in phase III clinical trials and for further steps) and the appropriate registration thereof. This aspect has been a crucial limiting factor for many public hospitals in recent years, as central health authorities progressively restrict the possibility of manufacturing and using cell-based treatments for in-house patients in the absence of standard product development and market authorization procedures [58]. Furthermore, due to the high risks and high costs of development, fewer industries have been investing in the development of ATMPs over the past years, despite the high scientific, clinical, and public interest [6,7,10,67]. 

Overall, it is clear that both the definition and use of a standardized process for HAC-based therapeutic product manufacture are of critical importance to all the involved stakeholders. Notwithstanding the regulatory requirements, the use of such systems is essential for the correct assessment of the safety, quality, and efficacy of an autologous cell-based product batch, which may by definition not be standardized on the account of inter-patient variability. Of equally high importance, such approaches are capital for the insurance of the appropriate transmission of technical knowledge and know-how, especially upon renewal of GMP manufacturing operators and of qualified personnel within the ad hoc institutional quality assurance system. 

### 4.6. Current GMP Manufacturing Process Limitations: The Open Question of HAC Cell Population Purity

A technical aspect of critical importance yet sub-optimally covered in many current clinical applications of HAC-based ACI consists in the assessment of the cultured cell population purity level. Specific tissue-related variability and the inclusion of a contaminating sub-population of cells other than HACs are possible at the time of the cartilage tissue biopsy harvest procedure, outlining the critical importance of the surgeon’s experience for the quality of the manufactured cellular APIs. Generally, methods for cell type identification and population purity assessment within processes of autologous chondrocyte manufacture constitute scientific and technical challenges. Such methods should necessarily provide a high level of confidence that the cellular APIs formulated in the finished cytotherapeutic product are of appropriate quality and purity, and that they are not composed of or contaminated by a different cell type to significant extents. To our knowledge and despite intense investigation, no specific genetic expression profile has been established and reported so far for the reliable characterization of cultured primary HACs. 

As regards cell population identification and cell population purity assessment, selected published reports have identified the usefulness of specific gene expression ratios to distinguish chondrocytes from synoviocytes or from dermal fibroblasts [68]. In their studies, Rapko et al. have analyzed in vitro monolayer cultures of these three types of adherent cells using transcriptome microarrays and RT-PCR [68]. It was shown that the gene encoding the MAGP2 protein was relatively more expressed in synoviocyte cultures and in dermal fibroblast cultures. Conversely, the authors have found that the gene encoding the HAPLN1 protein was relatively more expressed in chondrocyte cultures [68]. Therefore, it was shown that the use of quantitative RT-PCR analysis and of a logarithmic ratio of specific gene expression levels (i.e., HAPLN1:MAGP2) enabled the reliable identification of chondrocyte cultures for samples containing 65 ± 10% of chondrocytes in the analyzed cell population [68].

The analytic approach described hereabove was further applied by Asnaghi et al. for the specification of a threshold relative to primary chondrocyte cell population purity, within the process of using nasal chondrocytes for therapeutic ACI [69]. Based on these elements, the inclusion of the HAPLN1:MAGP2 ratio as a quality control assay (i.e., in parallel to the functional QC assays) in further optimized manufacturing processes is currently of highest interest, for the exclusion of HAC cultured cell population contamination by synoviocytes. In the presented case of therapeutic ACI in the CHUV, where the finished cytotherapeutic product is constituted by an HAC cell suspension, it could be argued that the cell population purity level is of key (but not critical) importance for the therapeutic process, as long as the function of the implanted cellular materials is demonstrated. This point would be substantiated by the potential therapeutic contribution of the synoviocyte fraction, combined with the HACs in a cell population characterized by imperfect population purity. However, precise or differential characterization of cell population purity and function would potentially be of higher importance in the case of seeding of the therapeutic cells on a multi-layer scaffold or a composite construct, where the resulting chondrogenic function may then be influenced by the cell population purity level of the considered cytotherapeutic API. 

In any case and despite the inherent limitations of cell population purity assessment (i.e., as presented for the use of HAPLN1:MAGP2 ratios), regulatory requirements will in all probability tend toward the specification of quantitative cut-off values or maximal synoviocyte population contamination levels for the release of manufactured cellular lots. Alternatively or in parallel, additional research could aim to prove, for defined cytotherapeutic applications that, within reasonable quantitative limits, a certain percentage of synoviocyte contamination does not alter the intended product function, as compared to a highly pure HAC population. Such considerations are to be applied to manufacturing processes, keeping in mind that the cell population purity for expanded HACs will in all probability not reach absolute purity, given the biopsy processing method and the absence of a specific cell selection step before the in vitro expansions. 

### 4.7. Technical and Clinical Future Directions: Cell Dose Considerations and Use of a Cell Scaffold for HAC-Based Therapeutic ACI in the CHUV

As regards the relative dose of therapeutic cells used in the referenced clinical trial (i.e., 2 × 10^6^ cells/cm^2^ of cartilage lesion), we should note that a preclinical cell dose adaptation study (i.e., for the assessment of the effects of smaller cell doses, notably) was not specifically previously performed in-house. As mentioned, the initial objective of importing the original technique of Brittberg et al. in Switzerland was to implement such cytotherapeutic approaches in the CHUV university hospital. This was performed by the authors while minimizing the regulatory workloads necessary before initiating the related clinical trial, building on the accumulated clinical experience and the hindsight of Prof. Brittberg et al. Notwithstanding and in view of new clinical trials in the CHUV, some cell dose optimization work has been performed in an in vitro preclinical setting, in view of optimizing the technological and manufacturing transposition to the in-house GMP manufacturing platform (results not shown). Generally, it is possible that lower cell doses may elicit appropriate therapeutic effects (i.e., especially for small cartilage lesions) in the clinic, yet due to the high variability between patients (i.e., epidemiological parameters and pathophysiological parameters), it is currently deemed best to maintain a cell dose (i.e., 2 × 10^6^ cells/cm^2^ of lesion), which may be assessed as relatively high but which is characterized by demonstrated extensive safety and efficacy hindsight. In other words, it is probably preferable to use a standard therapeutic cell dose higher than strictly necessary than to risk using a reduced cell dose resulting in sub-par clinical efficacy in a number of patients. 

Regarding the alternative and potential therapeutic use of uncultured autologous chondrocytes for cartilage repair promotion, some advantages may be clearly outlined (e.g., use of minimally manipulated materials, simplification of manufacturing requirements), as compared to the manufacturing processes described herein (Table S1). However, it is highly probable that the resort to multiple in vitro cell expansions is indeed preferable in the context of the therapeutic intervention of interest for two main technical reasons. Therein, the distinct in vitro manufacturing steps (i.e., including cell expansions and cell cryopreservation) allow for an extensive assessment of cellular lot quality with appropriate material liberation, as well as the obtention of sufficient cellular material quantities, starting with a minimal and standardized original cartilage biopsy size. Therefore, the rationale driving the use of in vitro cell expansions for cytotherapeutic material preparation is based mainly on the quality and the quantitative availability aspects of the therapeutic cellular materials, which are deemed overall to be critical for the obtention of consistent clinical success using such standardized transplant products. 

Furthermore, current considerations based on the available clinical experience with HAC-based injectable therapeutic products have led to the identification of an optimization potential regarding the cellular API delivery method. Various combinations of cellular APIs in suspension (e.g., Spherox^®^), cellular APIs in combination with ad hoc implantable scaffolds, or the use of acellular scaffolds for cartilage regeneration have been investigated, with necessary adaptations of the respective clinical and surgical protocols [70,71,72,73]. Among the available contenders for HAC-laden scaffolds, vehicles, and constructs are notably alginate gels, simple or complex collagen sheets, or rigid polymeric cell carriers [74,75,76,77,78,79,80,81]. A notable commercial example of such approaches, which relies on the use of porcine collagen membranes as a cell scaffold, is the MACI^®^ procedure (Vericel Corporation, Cambridge, MA, USA, www.maci.com, accessed on 14 February 2022).

Based on the technical possibility and on the functional interests of culturing HACs on a three-dimensional matrix or construct, such an approach is currently being investigated for the future in-house implementation of third-generation autologous chondrocyte transplants in clinical use in the CHUV. Numerous potential advantages are considered for the use of an implantable construct versus the current form of HAC suspensions, such as the enhanced function of the primary HACs, maintained in three-dimensional culture environments, the reduced losses of cells at the time of implantation, and the possibility of implanting the finished product by arthroscopy. Marketed matrices which would be adapted for such undertakings comprise the product Chondro-Gide^®^ (Geistlich Pharma, Wolhusen, Switzerland), which is currently being investigated for eventual GMP process transposition and clinical implementation in the context of a new prospective clinical trial for next-generation HAC-based cytotherapeutic care in the CHUV. 

## 5. Conclusions

The highly encouraging preliminary clinical results and the full technical success of autologous cytotherapeutic care provision within the context of the referenced ACI clinical trial in the CHUV have enabled a robust assessment of the overall process quality. Specifically, all 47 prescriptions of HAC-based cytotherapeutic products have in fine been followed by the timely delivery of liberated finished products meeting all predefined safety and quality requirements, clinician expectations, and patient needs. Despite the linear succession of events for biological material processing and HAC-based cytotherapeutic care provision, critical importance was outlined in this work for the effective multi-disciplinary collaboration and communication between the internal research and development, GMP manufacturing, and clinical orthopedic professional stakeholders within the considered Swiss healthcare institution. 

The data, processes, and related considerations presented in this study have contributed to facilitate both the approach and the transposition of the manufacture of HACs and of the related finished therapeutic products. By firstly retrospectively analyzing the available GMP manufacturing records for the currently produced HAC-based cytotherapeutic products, several technical aspects, such as the robustness of the production process were confirmed, along with some remaining technical margins of optimization. A quality assessment then enabled to identify key targets within the considered processes, for the continued improvement of cellular API GMP manufacturing (e.g., protocols for cell counting, cell cryopreservation) and the overall enhancement of cytotherapeutic product quality. Parallelly, important inter-individual variability existing between the patients was outlined, within the context of standardized transplant product preparation. Building on the aforementioned elements, parametric processes could be adapted for the considered therapeutic materials in view of further GMP manufacturing activities and additional clinical studies in the CHUV. Therein, standardized risk analysis-based process definition was performed, with specific focus set on process parameters, controls, targets, and acceptance criteria. Technological limitations of the overall process were also outlined (e.g., HAC cell population purity assessment, need for an implantable cell scaffold) and possible future directions of the work were explored, in a context of iterative and holistic quality amelioration. 

Notwithstanding the applicable regulatory requirements, the use of such specific GMP manufacturing systems is essential for the insurance of the appropriate safety, quality, and efficacy of an autologous cell-based product, which may by definition not be strictly standardized on the account of inter-patient variability. Overall, it was shown and discussed herein that the definition of standardized GMP manufacturing processes for cell-based therapeutic products is of critical importance for the clinical use and provision of high-quality ACI. While the cellular material GMP manufacturing process parameters and related targets may be very precisely defined, it is essential to constantly evaluate the related acceptance criteria, based on the analysis of all the available manufacturing data and records, to continuously maintain the high quality of clinical care and the ability to provide safe and efficient cytotherapeutic products. 

## Figures and Tables

**Table 1:** Overview of patient (*n* = 47) demographic data and injury specificities, related to autologous HAC therapeutic products manufactured for ACI treatment between September 2017 and December 2021. The data are presented as numerical averages, assorted with the corresponding standard deviations. ACI, autologous chondrocyte implantation; BMI, body mass index; HAC, human articular chondrocytes.

Patient Parameters		Average Numerical Data	
		Male Patients	Female Patients
Number of Patients (*n*)		29	18
Patient Age (years)		24.0 ± 7.5	24.2 ± 8.1
Patient BMI (kg/m^2^)		24.0 ± 4.3	22.0 ± 3.6
Lesion Size (cm^2^)		5.1 ± 2.4	4.1 ± 2.4
Number of Chondral Lesions (*n*)		12	13
Number of Osteochondral Lesions (*n*)		17	5
Affected Limb (Right/Left)		16/13	9/9
Anatomical Zone of the Lesion	Internal Femoral Condyle	14	6
	External Femoral Condyle	5	0
	Rotula	5	12
	Trochlea	3	0
	External Tibial Plateau	2	0

**Table 2:** Overview of the recorded GMP manufacturing process deviations for the 47 considered clinical HAC batches. Overall, a total of 8 deviations were qualified as minor, pertaining mostly to microbiological environmental monitoring or microbiological QC out-of-specification results for API manufacturing activities. Overall, a total of 5 deviations were qualified as major, pertaining mostly to biopsy transport conditions and to environmental microbiological monitoring out-of-specification results for finished product manufacturing activities. API, active pharmaceutical ingredients; GMP, good manufacturing practices; HAC, human articular chondrocytes; NA, non-applicable; QC, quality control; RNA, ribonucleic acid; TS, technical specifications.

Deviation Type	Number of Deviations	Description/Comments	Corrective Actions Resulting in Eventual Lot Liberation
Deviation to cell culture TS	8	Low number of cells in the preliminary cell population; cell seeding density variability; accelerated or delayed harvest due to inhomogeneous growth or to calendar constraints; low cell harvest yield; low cell viability at thawing	Communication and coordination with research laboratory and clinical unit personnel; documentary reconciliation of manufacturing records for all subsequent steps to demonstrate the appropriate endpoint quality of the material lots.
Deviation related to finished product TS	0	NA	NA
Deviations related to logistical process	3	Use of out-of-validity biopsy harvest and transport kit; biopsy transport in an alternative kit	Communication and coordination with research laboratory and clinical unit personnel resulting in conjoint validation of material use (risk analysis-based assessment, for sparing use of the valuable biospecimen); documentary reconciliation of manufacturing records for all subsequent steps to demonstrate the appropriate endpoint quality of the material lots; investigation of the origin and specifications of alternative and out-of-validity transport kits with clinical unit personnel.
Deviations related to manufacturing process controls ^1^	9	Airflow microbiological monitoring (*Methylobacterium radiotolerans*); imprint microbiological monitoring (*Bacillus* spp.; *Lysinibacillus fusiformis; Micrococcus luteus*; *Kocuria rhizophila*; unspecified Gram+ bacteria)	Complementary microbiological investigation performed on available retention samples.
Deviations related to storage process	0	NA	NA
Deviations related to microbiological QC ^2^	2	Cryotube microbiological testing (*Bacillus* spp.; *Moraxella osloensis* or *Enhydrobacter aerosaccus*)	Complementary microbiological QC & investigation performed on additional samples.
Deviations related to functional QC	2	Low RNA quantity	Repetition of the functional QC cell culture step for generation of appropriate biological material quantity.

^1^ Deviations related to the manufacturing process controls were in all cases related to monitoring (i.e., in-process controls) of particulates and contaminants in the class A production environment (i.e., sedimentation and contact-sampling). ^2^ Deviations related to microbiological QC comprised post-process controls for quality assessment of the manufactured material lots. However, out-of-specification microbiological assay results were not attributed to the material lots themselves, which were determined to be conform after repeat testing.

## Data Availability

Data presented in this study are available upon reasonable request made in writing to the corresponding authors.

## Supplementary Materials

The following are available online at https://www.mdpi.com/article/10.3390/cells11061016/s1, Figure S1: Schematic workflow for study nomenclature definition, Figure S2: Illustrated workflow for starting material procurement, Figure S3: Illustrated workflow for HAC API manufacturing, Figure S4: Illustrated workflow for HAC API functional quality control, Figure S5: Illustrated workflow for finished product manufacture, Table S1: Overview of autologous chondrocyte-based cytotherapeutic products used in clinical settings, Table S2: General RAM for sourcing, procurement, and culture initiation of primary HACs, Table S3: General RAM for banking of primary HACs, Table S4: Specific RAM for the microbiological quality of cultured HACs, Table S5: General RAM for HAC-based finished products, Supplementary Document “Process Parameters”: Tables SPP1–SPP4 in relation with Figure 7, Figure 8, Figure 9 and Figure 10 and describing details on the standardized and optimized parametric processes, Supplementary Document “Quality Attributes”: Tables SQA1 and SQA2 describing the proposed key and critical quality attributes of the HAC-based API and of the finished product, respectively.

## Abbreviations

ACI	autologous chondrocyte implantation
aHS API	autologous human serum active pharmaceutical ingredient
ASA	American Society of Anesthesiologists
ATIMP	advanced therapy investigational medicinal product
ATMP	advanced therapy medicinal product
cATMP	combined advanced therapy medicinal product
cGMP	current good manufacturing practices
CH	Helvetic Confederation
CHUV	centre hospitalier universitaire vaudois
CPC	cell production center
CPP	critical process parameter
CPR	Plastic and Reconstructive Surgery Service
CQA	critical quality attribute
DMSO	dimethyl sulfoxide
DNA	deoxyribonucleic acid
EC	European Commission
EDTA	ethylenediaminetetraacetic acid
EMA	European Medicines Agency
EU	European Union
FBS	fetal bovine serum
FDA	US Food and Drug Administration
GMP	good manufacturing practices
HAC	human articular chondrocytes
HCV	hepatitis C virus
HIV	human immunodeficiency virus
ICRS	International Cartilage Regeneration and Joint Preservation Society
IMPD	investigational medicinal product dossier
IPC	in-process control
KPP	key process parameter
KQA	key quality attribute
MCB	master cell bank
OTR	Orthopedics and Traumatology Service
Ph. Eur.	European pharmacopoeia
PPC	post-process control
PRP	platelet-rich plasma
QA	quality assurance
QC	quality control
RAM	risk analysis matrix
RH	relative humidity
RT-PCR	real-time polymerase chain reaction
TrSt	standardized transplant product
USA	United States of America
UTR	Regenerative Therapy Unit
WCB	working cell bank
