# Supplementary material for: Retrospective Analysis of Autologous Chondrocyte-Based Cytotherapy Production for Clinical Use: GMP Process-Based Manufacturing Optimization in a Swiss University Hospital
Source: Cells. 2022 Mar 17;11(6):1016. doi: 10.3390/cells11061016 (PMC8947208; doi:10.3390/cells11061016)
Supplement: Supplementary file 1 [file cells-11-01016-s001.zip › Process Parameters.pdf]

Supplementary Document – Process Parameters

Detailed parametric process definitions and specificities, relative to the standardized and optimized GMP manufacturing processes of HAC-based therapeutic products for ACI, are presented in this supplementary document "Process Parameters". Therein, each presented table (i.e., Tables SPP1–SPP4) respectively corresponds to a manuscript main figure (Figures 7–10). ACI, autologous chondrocyte implantation; GMP, good manufacturing practices; HAC, human articular chondrocytes.

Table SPP1. Detailed definition of KPPs and CPPs within the standardized parametric and controlled GMP process "Standardized Process: Biopsy ► Preliminary Cell Pool" presented in Figure 7. For each parameter item, the predefined targets and the IPCs or PPCs to be appropriately implemented are listed, along with the corresponding methods and acceptance criteria. CPP, critical process parameter; GMP, good manufacturing practices; h, hours; IPC, in-process control; KPP, key process parameter; min, minutes; Ph. Eur., European pharmacopoeia; PPC, post-process control; QA, quality assurance; RH, relative humidity.

CPP KPP	Parameter Definition	Process Targets (Cumulative)	Control Methods	IPCs PPCs	Acceptance Criteria (Cumulative)
CPP1	**Biological material identity**	Processing of the correct biological material, for the correct patient	Surgeon, operator & QA assessment	IPC: Batch file, prescription & transport tube label reconciliation	• Correspondence between production order form, batch file, and material labels
CPP2	**Biological material macroscopic qualification**	Biological material macroscopically qualified for in vitro enzymatic digestion, cell culture initiation & monolayer cell expansion	Surgeon & operator assessment	IPC: Biopsy dimensions & biopsy aspect	• Biopsy size > 3 mm × 10 mm<br>• Correct tissue isolated<br>• Adequate purity of isolated tissue
CPP3	**Sterility of procured biological materials / Processing**	No contaminating pathogens in procured biological starting materials & in biopsy processing environment	Bactec™ (transport medium & monitoring plates), Endosafe® (transport medium)	PPC: Microbiological testing & monitoring	• Absence of detection for specified and non-specified contaminants or values of detection < to specified thresholds
CPP4	**Sterility of the two-step enzymatic cell dissociation process from the biopsy**	No contaminating pathogens in the biopsy dissociation incubation vessel & in the processing environment	Bactec™ (digestion solution & monitoring plates)	PPC: Microbiological testing & monitoring	• Absence of detection for specified and non-specified contaminants or values of detection < to specified thresholds
CPP5	**Sterility of preliminary cell population / Processing**	No contaminating pathogens in the isolated preliminary cell population & in the processing environment	Bactec™ (cell suspension & monitoring plates)	PPC: Microbiological testing & monitoring	• Absence of detection for specified and non-specified contaminants or values of detection < to specified thresholds
CPP6	**Cellular viability of the preliminary cell population**	Cellular viability ≥ 75% upon enumeration after preliminary cell population enzymatic isolation	Ph. Eur. 2.7.29 [1]	IPC: Total & relative viable cell enumeration	• Cellular viability ≥ 75% upon enumeration after preliminary cell population isolation

KPP1	**Size of the minced tissue fragments after dissection**	Processed biological material of an appropriate size for in vitro enzymatic digestion & cell culture initiation	Operator assessment	IPC: Macroscopic assessment	• Individual biopsy fragment size < 1 mm^3
KPP2	**Humidification of the processed biological materials**	Maintenance of the processed biological materials in a humidified/liquid environment	Operator assessment	IPC: Macroscopic assessment	• No processing of the biopsy tissue & of tissue fragments in a dry state for > 3 min
KPP3	**Complete biopsy tissue fragment enzymatic digestion**	Complete biopsy tissue fragment digestion after the two-step enzymatic treatment with overnight incubation	Operator assessment	PPC: Macroscopic & microscopic assessment of digestion vessel	• No remanence of observable tissue fragments in the digestion vessel upon macroscopic & microscopic inspection
KPP4	**Total enzymatic reaction / Incubation time-period**	Total incubation time-period of the enzymatic digestion vessel, sufficient for complete tissue digestion but as short as possible	Operator assessment	IPC: Operator monitoring	• Total incubation time-period of the enzymatic digestion vessel of > 12 h and < 24 h
KPP5	**Incubation parameter stability for the enzymatic tissue digestion reaction**	Maintenance of adequate incubation conditions throughout the enzymatic reaction	Incubator monitoring system records, operator assessment	IPC: Incubator monitoring & display systems<br>PPC: Incubator monitoring system data logs	• Relative limits [2] of 37 °C ± 2 °C, 10% ± 1% CO_2, 85% ± 5% RH in the incubator<br>• Absolute limits [3] of 34 °C ± 4 °C, 0% – 10% CO_2, 0% – 95% RH in the incubator
KPP6	**Total cell quantity in the preliminary cell population**	Maximization of the cell quantity in the preliminary cell population	Ph. Eur. 2.7.29 [1]	IPC (*redundant with CPP6*): Total cell enumeration	• Total cell quantity > 1.5 × 10^5 cells in the isolated preliminary cell population

[1] Ph. Eur. method 2.7.29 "Nucleated cell count and viability". [2] Relative limits refer to the successive incubation phases between the cell culture initiation, the culture medium exchanges, and the cell harvest procedures (i.e., intermittent incubation time-periods characterized by closed incubator doors). [3] Absolute limits refer to the overall cell culture maintenance timeframe, from the initial culture incubation up to the cell harvest procedure (i.e., total and continuous incubation time-period considering incubator door opening for cell culture handling).

Table SPP2. Detailed definition of KPPs and CPPs within the standardized parametric and controlled GMP process "Standardized Process: Preliminary Cell Pool ► MCB" presented in Figure 8. For each parameter item, the predefined targets and the IPCs or PPCs to be appropriately implemented are listed, along with the corresponding methods and acceptance criteria. CPP, critical process parameter; GMP, good manufacturing practices; h, hours; IPC, in-process control; KPP, key process parameter; MCB, master cell bank; min, minutes; NAT, nucleic acid amplification technique; Ph. Eur., European pharmacopoeia; PPC, post-process control; QA, quality assurance; QC, quality control; RH, relative humidity.

CPP<br>KPP	Parameter Definition	Process Targets (Cumulative)	Control Methods	IPCs<br>PPCs	Acceptance Criteria (Cumulative)
CPP1	**Biological material identity**	Processing of the correct biological materials	Operator assessment	IPC: Batch file & culture vessel label reconciliation	• Correspondence between the batch file and the culture vessel labels
CPP2	**Cell adherence for in vitro monolayer culture**	Adherence of ≥ 70% of the seeded cells after 24 h of incubation following cell seeding	Operator assessment by contrast phase microscopy	IPC: Microscopic monitoring of monolayer cultures	• Adherence of ≥ 70% of the seeded cells after 24 h of incubation following cell seeding
CPP3	**Cell proliferation during in vitro monolayer culture**	• Confirmation of positive cell confluency level evolution between medium exchanges<br>• Confirmation of proliferative cellular behaviour in culture	Operator assessment by contrast phase microscopy, photographic recording	IPC: Microscopic monitoring at medium exchange, photographic recording of cultures	• Confirmation of proliferative cellular morphology & behaviour in culture<br>• (Confluency value at medium exchange X) ≥ (Confluency value at medium exchange X-1)
CPP4	**Identity/purity of the cultured cell population**	• Specific cellular morphology & behaviour maintenance in monolayer culture<br>• Absence of multiple cell populations in monolayer culture	Operator assessment by contrast phase microscopy, photographic recording, NAT	IPC (*redundant with CPP3*): Microscopic monitoring during monolayer expansion, photographic recording of cultures<br>PPC: Functional/Identity QC	• Spindle-shaped cells; distinctive fibroblastic cellular phenotype in culture<br>• Absence of multiple observable cell populations in culture<br>• Chondrogenic gene expression levels induced in functional QC<br>• Gene expression ratios within historical data brackets for identity QC
CPP5	**Sterility of in vitro cell culture conditions / Processing**	No contaminating pathogens in the cultured biological materials & in the processing environment	Bactec™ (culture medium & monitoring plates)	PPC: Microbiological testing & monitoring	• Absence of detection for specified and non-specified contaminants or values of detection < to specified thresholds
CPP6	**Cell viability after endpoint enzymatic harvest**	Cellular viability ≥ 85% upon endpoint enzymatic harvest	Ph. Eur. 2.7.29 [1]	IPC: Total & viable cell enumeration after enzymatic harvest	• Cellular viability ≥ 85% upon endpoint enzymatic harvest

CPP7	**Sterility of the conditioned MCB cell lot**	No contaminating pathogens in the conditioned MCB cell lot & in the processing environment	Bactec™ (culture medium & monitoring plates), Endosafe® (cell rinsing solution)	PPC: Microbiological testing & monitoring	• Absence of detection for specified and non-specified contaminants or values of detection < to specified thresholds
CPP8	**Cryopreserved material stability in storage**	• No deterioration of material primary conditioning system (cryotubes)<br>• Non-critical loss of biological material viability & functionality	Operator assessment, Ph. Eur. 2.7.29 [1], chondrogenic gene expression assessment	PPC: Material inspection, qualitative & functional QCs	• No deterioration of cryotube integrity<br>• Cell viability ≥ 70% upon thawing<br>• Chondrogenic activity within historical data brackets
CPP9	**Cryogenic storage system stability**	No structural ruptures of storage system, catastrophic drop in liquid nitrogen level, or catastrophic rise in system temperature	Operator assessment & alarm systems	IPC: Storage tank temperature & level monitoring & displays<br>PPC: Storage tank data logs	• Maintenance of storage system structural integrity<br>• Storage tank temperature constantly ≤ –145 °C<br>• Liquid nitrogen level constantly above the lower critical level
CPP10	**Documentary & material traceability**	Availability of all relevant authenticated documents & records for cell type master file, biobank inventory, and manufacturing batch files at the time of reconciliation	Operator & QA assessment	IPC/PPC: Follow-up of manufacturing data in cell type file & in biobank inventory	• Availability of all relevant authenticated documents & records for cell type master file, biobank inventory, and manufacturing batch files at the time of reconciliation
KPP1	**Overall cell culture manipulation time**	Minimization of the time-period during which the cells are not in adherent state	Operator assessment	IPC: Total time the cells are processed in non-adherent state	• Total time the cells are processed in non-adherent state of < 75 min
KPP2	**Cell culture homogeneity in monolayer culture**	Homogenous growth of the cell monolayer over the available culture surfaces	Operator assessment by contrast phase microscopy, photographic recording	IPC (*redundant with CPP3*): Microscopic monitoring & photographic recording during the expansion	• Absence of unpopulated cell culture surface of ≥ 15% of the total available cell culture surface in each culture vessel
KPP3	**Cell metabolism during the monolayer expansion**	Maintenance of bright red to dark red colouring of the cell culture medium containing a phenol red pH indicator	Operator macroscopic assessment	IPC: Macroscopic monitoring of cell culture medium aspect	• Absence of orange or purple colouring of the cell culture medium and of signs of severe cell starvation

KPP4	**Incubation parameter stability for cell expansion**	Maintenance of adequate incubation conditions throughout the culture expansions	Monitoring system records, operator assessment	IPC: Incubator monitoring & display systems<br>PPC: Incubator monitoring system data logs	• Relative limits [2] of 37 °C ± 2 °C, 10% ± 1% CO_2, 85% ± 5% RH in the incubator<br>• Absolute limits [3] of 34 °C ± 4 °C, 0% – 10% CO_2, 0% – 95% RH in the incubator
KPP5	**Total culture time-period for monolayer cell expansion**	Consistent preliminary cell culture times, within historical data brackets	Operator assessment	IPC: Monitoring during in vitro cell expansion	• Harvest of the expanded preliminary cell population at 11 ± 3 days of culture<br>• Harvest of the expanded cell seed at 5 ± 3 days of culture
KPP6	**Cell confluency level at endpoint enzymatic harvest**	Maximized cell confluency level at the endpoint harvest	Operator assessment by contrast phase microscopy, photographic recording	IPC (*redundant with CPP3*): Microscopic monitoring of cultures, photographic recording of cultures	• Cell confluency level at the time of harvest > 60% in each cell culture vessel
KPP7	**Cell quantity at endpoint enzymatic harvest**	Maximization of the manufacturing cell yield	Ph. Eur. 2.7.29 [1]	IPC (*redundant with CPP6*): Total cell enumeration	• Cell quantity at harvest > 2.5 × 10^6 cells for the expanded preliminary cell population<br>• Cell quantity at harvest > 25 × 10^6 cells for the expanded cell seed population
KPP8	**Cell quantity available for conditioning of the MCB lot**	Maximization of the MCB lot size	Ph. Eur. 2.7.29 [1]	IPC (*redundant with CPP6*): Total cell enumeration & reconciliation	• Cell quantity at harvest > 25 × 10^6 cells for conditioning of the MCB lot
KPP9	**Rate of conditioned MCB lot primary freezing**	Rate of cooling of –1 °C/min from ambient temperature to –80 °C during the primary freezing phase	Operator assessment	PPC: Freezer temperature logs	• Rate of cooling of –1 °C/min from ambient temperature to –80 °C
KPP10	**Time to transfer of the MCB lot to liquid nitrogen storage**	Transfer to liquid nitrogen storage after sufficient primary freezing period but < 24 h after primary freezing initiation	Operator assessment	IPC: Operator monitoring	• Transfer to liquid nitrogen storage > 4 h after primary freezing initiation but < 24 h after primary freezing initiation

KPP11	**Overall batch manipulation time**	Minimization of the time-period between confluent cell population harvest & conditioned MCB cell lot cryogenic storage	Operator assessment	IPC: Total time the cells are processed before cryogenic storage	• Transfer to liquid nitrogen storage < 36 h after cell population harvest initiation

[1] Ph. Eur. method 2.7.29 "Nucleated cell count and viability".[2] Relative limits refer to the successive incubation phases between the cell culture initiation, the culture medium exchanges, and the cell harvest procedures (i.e., intermittent incubation time-periods characterized by closed incubator doors). [3] Absolute limits refer to the overall cell culture maintenance timeframe, from the initial culture incubation up to the cell harvest procedure (i.e., total and continuous incubation time-period considering incubator door opening for cell culture handling).

Table SPP3. Detailed definition of KPPs and CPPs within the optimized parametric and controlled GMP process "Optimized Cell Banking Process: MCB ► WCB" presented in Figure 9. For each parameter item, the predefined targets and the IPCs or PPCs to be appropriately implemented are listed, along with the corresponding methods and acceptance criteria. CPP, critical process parameter; GMP, good manufacturing practices; h, hours; IPC, in-process control; KPP, key process parameter; MCB, master cell bank; min, minutes; NAT, nucleic acid amplification technique; Ph. Eur., European pharmacopoeia; PPC, post-process control; QA, quality assurance; QC, quality control; RH, relative humidity; WCB, working cell bank.

CPP<br>KPP	Parameter Definition	**Process Targets** (Cumulative)	Control Methods	IPCs<br>PPCs	**Acceptance Criteria** (Cumulative)
CPP1	**Biological material identity**	Processing of the correct biological material	Operator assessment	IPC: Batch file & culture vessel label reconciliation	• Correspondence between the batch file and the culture vessel labels
CPP2	**Cell adherence for in vitro monolayer culture**	Adherence of ≥ 70% of seeded cells after 24 h of incubation following cell seeding	Operator assessment by contrast phase microscopy	IPC: Microscopic monitoring of monolayer cultures	• Adherence of ≥ 70% of seeded cells after 24 h of incubation following cell seeding
CPP3	**Cell proliferation during in vitro monolayer culture**	• Confirmation of positive cell confluency level evolution between medium exchanges<br>• Confirmation of proliferative cellular behaviour in culture	Operator assessment by contrast phase microscopy, photographic recording	IPC: Microscopic monitoring at medium exchange, photographic recording of cultures	• Confirmation of proliferative cellular behaviour in culture<br>• (Confluency value at medium exchange X) ≥ (Confluency value at medium exchange X-1)
CPP4	**Identity/purity of the cultured cell population**	• Specific cellular morphology & behaviour maintenance in monolayer culture<br>• Absence of multiple cell populations in monolayer culture	Operator assessment by contrast phase microscopy, photographic recording, NAT	IPC (*redundant with CPP3*): Microscopic monitoring during monolayer expansion, photographic recording of cultures<br>PPC: Functional/Identity QC	• Spindle-shaped cells; distinctive fibroblastic cellular phenotype in culture<br>• Absence of multiple observable cell populations in culture<br>• Chondrogenic gene expression levels induced in functional QC<br>• Gene expression ratios within historical data brackets for identity QC
CPP5	**Sterility of in vitro cell culture conditions / Processing**	No contaminating pathogens in the cultured biological materials & in the processing environment	Bactec™ (culture medium & monitoring plates)	PPC: Microbiological testing & monitoring	• Absence of detection for specified and non-specified contaminants or values of detection < to specified thresholds
CPP6	**Cell viability after endpoint enzymatic harvest**	Cellular viability ≥ 85% upon endpoint enzymatic harvest	Ph. Eur. 2.7.29 [1]	IPC: Total & viable cell enumeration after enzymatic harvest	• Cellular viability ≥ 85% upon enzymatic harvest

CPP7	**Cell type quality/safety**	• Cell type of sufficient quality for pharmaceutical use<br>• Non-tumorigenic cell type	Operator assessment, soft agar colony formation assays	PPC: Assessment of tumorigenic potential	• Absence of detected tumorigenic potential of the primary cell type
CPP8	**Cell type in vitro stability**	• Sufficient stability for serial in vitro cell culture expansion of the primary cell type of interest<br>• No significant genetic instability of the cultured materials	Operator assessment, cell karyotyping	IPC: Cell type lifespan & cell karyotyping analysis	• In vitro lifespan of the primary cell type ≥ 4 passages<br>• No significant genetic instability reported
CPP9	**Sterility of the conditioned WCB cell lot**	No contaminating pathogens in the conditioned WCB cell lot & in the processing environment	Bactec™ (culture medium & monitoring plates), Endosafe® (cell rinsing solution)	PPC: Microbiological testing & monitoring	• Absence of detection for specified and non-specified contaminants or values of detection < to specified thresholds
CPP10	**Cryopreserved material stability in storage**	• No deterioration of material primary conditioning system (cryotubes)<br>• Non-critical loss of biological material viability and functionality	Operator assessment, Ph. Eur. 2.7.29 [1], chondrogenic gene expression assessment	PPC: Material inspection, qualitative & functional QCs	• No deterioration of cryotube integrity<br>• Cell viability ≥ 70% upon thawing<br>• Chondrogenic activity within historical data brackets
CPP11	**Cryogenic storage system stability**	No structural ruptures of storage system, catastrophic drop in liquid nitrogen level, or catastrophic rise in system temperature	Operator assessment & alarm systems	IPC: Storage tank temperature & level monitoring & displays<br>PPC: Storage tank data logs	• Maintenance of storage system structural integrity<br>• Storage tank temperature constantly ≤ –145 °C<br>• Nitrogen level constantly above the lower critical level
CPP12	**Documentary & material traceability**	Availability of all relevant authenticated documents & records for cell type master file, biobank inventory, and manufacturing batch files at the time of reconciliation	Operator & QA assessment	IPC/PPC: Follow-up of manufacturing data in cell type file & in biobank inventory	• Availability of all relevant authenticated documents & records for cell type master file, biobank inventory, and manufacturing batch files at the time of reconciliation
KPP1	**Rate of vial thawing for initiation from storage**	Rapid thawing of frozen vials	Operator assessment	IPC: Operator monitoring	• Complete thawing of the vials < 10 min after removal from liquid nitrogen storage and < 5 min after incubation at 37 °C

KPP2	**Overall cell culture manipulation time**	Minimization of the time-period during which the cells are not in adherent state	Operator assessment	IPC: Total time the cells are processed in non-adherent state	• Total time the cells are processed in non-adherent state of < 75 min
KPP3	**Cell culture homogeneity in monolayer culture**	Homogenous growth of the cell monolayer over the available cell culture surfaces	Operator assessment by contrast phase microscopy, photographic recording	IPC (*redundant with CPP3*): Microscopic monitoring & recording during expansion	• Absence of unpopulated cell culture surface of ≥ 15% of the total available cell culture surface in each culture vessel
KPP4	**Cell metabolism during monolayer expansion**	Maintenance of bright red to dark red colouring of the cell culture medium containing a phenol red indicator	Operator macroscopic assessment	IPC: Macroscopic monitoring of cell culture medium aspect	• Absence of orange or purple colouring of the cell culture medium or signs of severe cell starvation
KPP5	**Incubation parameter stability for cell expansion**	Maintenance of adequate incubation conditions throughout the cell culture expansions	Monitoring system records, operator assessment	IPC: Incubator monitoring & display systems<br>PPC: Incubator monitoring system data logs	• Relative limits [2] of 37 °C ± 2 °C, 10% ± 1% CO_2, 85% ± 5% RH in the incubator<br>• Absolute limits [3] of 34 °C ± 4 °C, 0% – 10% CO_2, 0% – 95% RH in the incubator
KPP6	**Total culture time-period for monolayer cell expansion**	Consistent preliminary culture times, within historical data brackets	Operator assessment	IPC: Monitoring during in vitro cell expansion	• Harvest of expanded cell population at 5 ± 3 days of culture
KPP7	**Cell confluency level at endpoint enzymatic harvest**	Maximized cell confluency level at endpoint enzymatic harvest	Operator assessment by contrast phase microscopy, photographic recording	IPC (*redundant with CPP3*): Microscopic monitoring of cultures, photographic recording of cultures	• Cell confluency level at the time of endpoint enzymatic harvest > 60% in each culture vessel
KPP8	**Cell quantity at endpoint enzymatic harvest**	Maximization of manufacturing cell yield	Ph. Eur. 2.7.29 [1]	IPC (*redundant with CPP6*): Total cell enumeration	• Cell quantity at harvest > 15 × 10^6 cells for the expanded cell population

KPP9	**Cell quantity available for conditioning of the WCB lot**	Maximization of the WCB lot size	Ph. Eur. 2.7.29 [1]	IPC (*redundant with CPP6*): Total cell enumeration & reconciliation	• Cell quantity at harvest > 15 × 10^6 cells for conditioning of the WCB lot
KPP10	**Rate of conditioned WCB lot primary freezing**	Rate of cooling of −1 °C/min from ambient temperature to −80 °C during the primary freezing phase	Operator assessment	PPC: Freezer temperature logs	• Rate of cooling of −1 °C/min from ambient temperature to −80 °C
KPP11	**Time to transfer of the WCB lot to liquid nitrogen storage**	Transfer to liquid nitrogen storage after sufficient primary freezing period but < 24 h after primary freezing initiation	Operator assessment	IPC: Operator monitoring	• Transfer to liquid nitrogen storage > 4 h after primary freezing initiation but < 24 h after primary freezing initiation
KPP12	**Overall cell batch manipulation time**	Minimization of the time-period between confluent cell population harvest & conditioned WCB cell lot cryogenic storage	Operator assessment	IPC: Total time the cells are processed before cryogenic storage	• Transfer to liquid nitrogen storage < 36 h after cell population harvest initiation

[1] Ph. Eur. method 2.7.29 "Nucleated cell count and viability".[2] Relative limits refer to the successive incubation phases between the cell culture initiation, the culture medium exchanges, and the cell harvest procedures (i.e., intermittent incubation time-periods characterized by closed incubator doors). [3] Absolute limits refer to the overall cell culture maintenance timeframe, from the initial culture incubation up to the cell harvest procedure (i.e., total and continuous incubation time-period considering incubator door opening for cell culture handling).

Table SPP4. Detailed definition of KPPs and CPPs within the standardized parametric and controlled GMP process "Standardized Process: MCB/WCB ► Finished Product" presented in Figure 10. For each process item, the predefined targets and the IPCs or PPCs to be appropriately implemented are listed, along with the corresponding methods and acceptance criteria. CPP, critical process parameter; GMP, good manufacturing practices; h, hours; IPC, in-process control; KPP, key process parameter; MCB, master cell bank; NAT, nucleic acid amplification technique; Ph. Eur., European pharmacopoeia; PPC, post-process control; QA, quality assurance; QC, quality control; RH, relative humidity; WCB, working cell bank.

CPP KPP	Parameter Definition	Process Targets (Cumulative)	Control Methods	IPCs PPCs	Acceptance Criteria (Cumulative)
CPP1	**Biological material identity**	Processing of the correct biological material	Operator & QA assessment	IPC: Batch file & label reconciliation	• Correspondence between the batch file and the material labels
CPP2	**Cell adherence for in vitro monolayer culture**	Adherence of ≥ 70% of the seeded cells after 24 h of incubation following cell seeding	Operator assessment by contrast phase microscopy	IPC: Microscopic monitoring of monolayer cultures	• Adherence of ≥ 70% of seeded cells after 24 h of incubation following cell seeding
CPP3	**Cell proliferation during in vitro monolayer culture**	• Confirmation of positive confluency evolution between medium exchanges<br>• Confirmation of proliferative cellular behaviour in culture	Operator assessment by contrast phase microscopy, photographic recording	IPC: Microscopic monitoring at medium exchange, photographic recording of cultures	• Confirmation of proliferative cellular behaviour in culture<br>• (Confluency value at medium exchange X) ≥ (Confluency value at medium exchange X-1)
CPP4	**Identity/purity of the cultured cell population**	• Specific cellular morphology & behaviour maintenance in monolayer culture<br>• Absence of multiple cell populations in monolayer culture	Operator assessment by contrast phase microscopy, photographic recording, NAT	IPC (*redundant with CPP3*): Microscopic monitoring during monolayer expansion, photographic recording of cultures<br>PPC: Functional/Identity QC	• Spindle-shaped cells; distinctive fibroblastic cellular phenotype in culture<br>• Absence of multiple observable cell populations in culture<br>• Chondrogenic gene expression levels induced in functional QC<br>• Gene expression ratios within historical data brackets for identity QC
CPP5	**Sterility of in vitro cell culture conditions / Processing**	No contaminating pathogens in the cultured biological materials & in the processing environment	Bactec™ (culture medium & monitoring plates)	PPC: Microbiological testing & monitoring	• Absence of detection for specified and non-specified contaminants or values of detection < to specified thresholds
CPP6	**Cell viability after endpoint enzymatic harvest**	Cellular viability ≥ 85% upon endpoint enzymatic harvest	Ph. Eur. 2.7.29 [1]	IPC: Total & viable cell enumeration after enzymatic harvest	• Cellular viability ≥ 85% upon endpoint enzymatic harvest

CPP7	**Biological material identity**	Processing of the correct biological material, for the correct patient	Operator & QA assessment	IPC: Batch file & label reconciliation	• Correspondence between the batch file and the material labels
CPP8	**Finished product sterility**	No contaminating pathogens in the finished product	Bactec™ & Endosafe® (culture medium & cell rinsing solution)	PPC: Microbiological testing	• Absence of detection for specified and non-specified contaminants or values of detection < to specified thresholds
CPP9	**Cell viability in the finished product**	Cellular viability ≥ 85% upon finished product formulation	Ph. Eur. 2.7.29 [1]	IPC (*redundant wit CPP6*): Viable cell enumeration	• Cellular viability ≥ 85% upon finished product formulation
CPP10	**Cell quantity in the finished product**	Cell quantity in the finished product of 3×10^4 cells/μL	Ph. Eur. 2.7.29 [1]	IPC (*redundant with CPP6*): Total cell enumeration at harvest	• Cell quantity in the finished product of 3×10^4 cells/μL
CPP11	**Finished product batch uniformity**	Uniformity of the conditioned finished product batch	Operator assessment	PPC: Qualitative QC	• Overall and detailed appreciation within historical data brackets<br>• Equality of conditioned product volumes in the case of multiple syringes
CPP12	**Documentary & material traceability**	Availability of all relevant authenticated documents & records for cell type master file, biobank inventory, and manufacturing batch files at the time of reconciliation	Operator & QA assessment	IPC/PPC: Follow-up of manufacturing data in cell type file & in biobank inventory	• Availability of all relevant authenticated documents & records for cell type master file, biobank inventory, and manufacturing batch files at the time of reconciliation
KPP1	**Rate of vial thawing for initiation from storage**	Rapid thawing of frozen vials	Operator assessment	IPC: Operator monitoring	• Complete thawing of the vials < 10 min after removal from liquid nitrogen storage and < 5 min after incubation at 37 °C
KPP2	**Overall cell culture manipulation time**	Minimization of the time-period during which the cells are not in adherent state	Operator assessment	IPC: Total time the cells are processed in non-adherent state	• Total time the cells are processed in non-adherent state of < 75 min

KPP3	**Cell culture homogeneity in monolayer culture**	Homogenous growth of the cell monolayer over the available cell culture surfaces	Operator assessment by contrast phase microscopy, photographic recording	IPC (*redundant with CPP3*): Microscopic monitoring & recording during expansion	• Absence of unpopulated cell culture surface of ≥ 15% of the total available cell culture surface in each culture vessel
KPP4	**Cell metabolism during monolayer expansion**	Maintenance of bright red to dark red colouring of the culture medium containing a phenol red indicator	Operator macroscopic assessment	IPC: Macroscopic monitoring of cell culture medium aspect	• Absence of orange or purple colouring of the cell culture medium or signs of severe cell starvation
KPP5	**Incubation parameter stability for cell expansion**	Maintenance of adequate incubation conditions throughout the cell culture expansions	Monitoring system records, operator assessment	IPC: Incubator monitoring & display systems<br>PPC: Incubator monitoring system data logs	• Relative limits [2] of 37 °C ± 2 °C, 10% ± 1% CO_2, 85% ± 5% RH in the incubator<br>• Absolute limits [3] of 34 °C ± 4 °C, 0% – 10% CO_2, 0% – 95% RH in the incubator
KPP6	**Total culture time-period for monolayer cell expansion**	Consistent preliminary culture times, within historical data brackets	Operator assessment	IPC: Monitoring during in vitro cell expansion	• Harvest of expanded cell population at 7 ± 3 days of culture
KPP7	**Cell confluency level at endpoint enzymatic harvest**	Maximized cell confluency level at endpoint enzymatic harvest	Operator assessment by contrast phase microscopy, photographic recording	IPC (*redundant with CPP3*): Microscopic monitoring of cultures, photographic recording of cultures	• Cell confluency level at the time of endpoint enzymatic harvest > 60% in each culture vessel
KPP8	**Cell quantity at endpoint enzymatic harvest**	Maximization of manufacturing cell yield	Ph. Eur. 2.7.29 [1]	IPC (*redundant with CPP6*): Total cell enumeration	• Cell quantity at endpoint enzymatic harvest sufficient for the manufacture of the finished product lot
KPP9	**Finished product storage temperature stability**	Stability of the finished product transport & storage temperatures	Operator & QA assessment	PPC: Transport container temperature logs	• Stable transport container temperature at 15–30 °C

[1] Ph. Eur. method 2.7.29 ”Nucleated cell count and viability”.[2] Relative limits refer to the successive incubation phases between the cell culture initiation, the culture medium exchanges, and the cell harvest procedures (i.e., intermittent incubation time-periods characterized by closed incubator doors). [3] Absolute limits refer to the overall cell culture maintenance timeframe, from the initial culture incubation up to the cell harvest procedure (i.e., total and continuous incubation time-period considering incubator door opening for cell culture handling).
