# Supplementary material for: Retrospective Analysis of Autologous Chondrocyte-Based Cytotherapy Production for Clinical Use: GMP Process-Based Manufacturing Optimization in a Swiss University Hospital
Source: Cells. 2022 Mar 17;11(6):1016. doi: 10.3390/cells11061016 (PMC8947208; doi:10.3390/cells11061016)
Supplement: Supplementary file 1 [file cells-11-01016-s001.zip › Quality Attributes.pdf]

## Supplementary Document – Quality Attributes

Key and critical quality attributes of the cellular API and of the finished HAC-based therapeutic product are presented in tabular form (i.e., Tables SQA1 and SQA2, respectively) in this supplementary document "Quality Attributes". API, active pharmaceutical ingredient; HAC, human articular chondrocyte.

**Table SQA1.** Established cryopreserved cellular API (i.e., cultured HACs) quality attributes, which were specified as KQA or as CQA. API, active pharmaceutical ingredient; CQA, critical quality attribute; EU, endotoxin units; HAC, human articular chondrocyte; KQA, key quality attribute.

| **API Quality Attributes** | **Attribute Type** | **Requirements for an API Lot** (Cumulative) |
|---|---|---|
| • **Cellular API Identity** | CQA | Appropriate cellular morphology and behaviour in two-dimensional culture, appropriate increase in chondrogenic gene expression in three-dimensional culture, appropriate specific gene expression markers. |
| • **Cellular API Purity** | KQA | Appropriate cellular morphology and behaviour in two-dimensional culture, appropriate specific gene expression markers. |
| • **Cellular API Function** | CQA | Appropriate increase in chondrogenic gene expression in three-dimensional culture. |
| • **Sterility (bacteria and fungi)** | CQA | Absence of detection for specified and non-specified contaminants or values of detection < to specified thresholds. |
| • **Sterility (mycoplasma)** | CQA | Absence of detection for specified and non-specified contaminants. |
| • **Acceptable Endotoxin Level** | CQA | Endotoxin level < 0.2 EU/mL. |
| • **Cellular Viability Maintenance** | CQA | Cellular viability maintenance throughout storage resulting in cellular viability of > 85% upon initiation from storage. |
| • **Cellular Proliferation Capacity Maintenance** | CQA | Possibility of in vitro monolayer adherence and expansion for ≥ 3 passages. |
| • **Number of Cells/API Container** | KQA | Specified number of cells/container ± 20%. |
| • **API Identification** | CQA | Correct labelling of API packaging materials. |
| • **Appropriate API Storage** | CQA | Cryogenic API storage at temperatures constantly < −145 °C. |
| • **Appropriate API Validity** | CQA | Use of the API within the validated API validity period. |

**Table SQA2.** Established HAC-based injectable therapeutic product quality attributes, which were specified as KQA or as CQA. API, active pharmaceutical ingredient; CQA, critical quality attribute; EU, endotoxin units; HAC, human articular chondrocyte; KQA, key quality attribute.

| **Finished Product Quality Attributes** | **Attribute Type** | **Requirements for a Finished Product Lot** (Cumulative) |
|---|---|---|
| • **Formulated API Identity** | **CQA** | Appropriate traceability for formulation of the correct API in the finished product. |
| • **Sterility (bacteria and fungi)** | **CQA** | Absence of detection for specified and non-specified contaminants or values of detection < to specified thresholds. |
| • **Sterility (mycoplasma)** | **CQA** | Absence of detection for specified and non-specified contaminants. |
| • **Acceptable Endotoxin Level** | **CQA** | Endotoxin level < 0.2 EU/mL. |
| • **Cellular Viability Maintenance** | **CQA** | Cellular viability > 75% in the finished product during the whole validated product validity period. |
| • **Finished Product Tonicity & pH** | **KQA** | Appropriate product tonicity and pH for the considered administration route. |
| • **Total Cell Dose** | **KQA** | Specified number of cells/product dose ± 20%. |
| • **Product Quantity** | **KQA** | Appropriate product quantity at the time of reconciliation with the medical prescription. |
| • **Product Administration System** | **CQA** | Appropriate administration system for the planned surgical operation, as prescribed. |
| • **Product Identification** | **CQA** | Correct labelling of product packaging materials and product primary container. |
| • **Appropriate Product Storage** | **CQA** | Ambient temperature storage of the finished product. |
| • **Appropriate Product Validity** | **CQA** | Use of the finished product within the validated product validity period. |
