# Supplementary material for: Retrospective Analysis of Autologous Chondrocyte-Based Cytotherapy Production for Clinical Use: GMP Process-Based Manufacturing Optimization in a Swiss University Hospital
Source: Cells. 2022 Mar 17;11(6):1016. doi: 10.3390/cells11061016 (PMC8947208; doi:10.3390/cells11061016)
Supplement: Supplementary file 1 [file cells-11-01016-s001.zip › Supplementary Figures.pdf]

Supplementary Figures

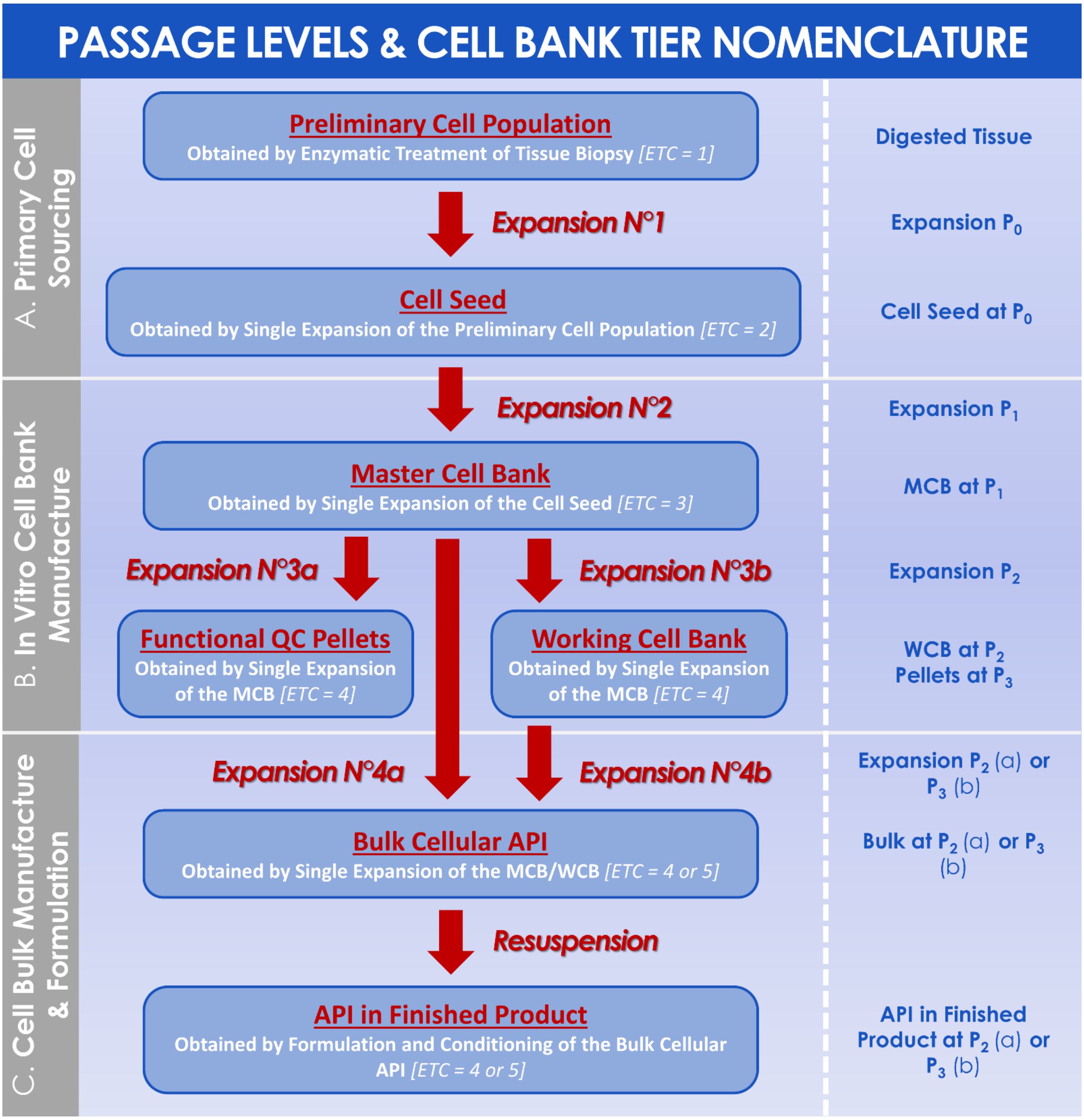

Figure S1. Schematic workflow for specification of the in vitro cell passage level and of the cell bank nomenclature within the HAC API and the finished product manufacturing activities, as described in the standardized and optimized GMP parametric processes in the second part of the study (Figures 7–10). The overall process is presented in three distinct steps, namely the primary cell sourcing (**A**), the in vitro cell bank manufacture (**B**), and the cell bulk manufacture and formulation (**C**). It is to note that, for the considered processes and for others used in the CHUV university hospital, the cell passage level nomenclature is preferred over the cell population doubling values to describe the in vitro cell type age. This allows to take into account the number of enzymatic treatments (i.e., stress for the cells) necessary to dissociate the cells from the tissue biopsy and then from the culture vessel surface. The use of the cell passage level nomenclature is justified therein by the use of defined and consistent relative cell seeding densities, cell culture time-periods, and cell confluency levels at harvest during the in vitro manufacturing steps comprising cellular expansions. An equivalence table may be further established as appropriate between the specific cell passage levels and the cell population doubling value ranges which characterize the cell populations at each step of the manufacturing process. API, active pharmaceutical ingredient; CHUV, centre hospitalier universitaire vaudois; ETC, enzymatic treatment count; GMP, good manufacturing practices; HAC, human articular chondrocyte; MCB, master cell bank; P, passage level; WCB, working cell bank.

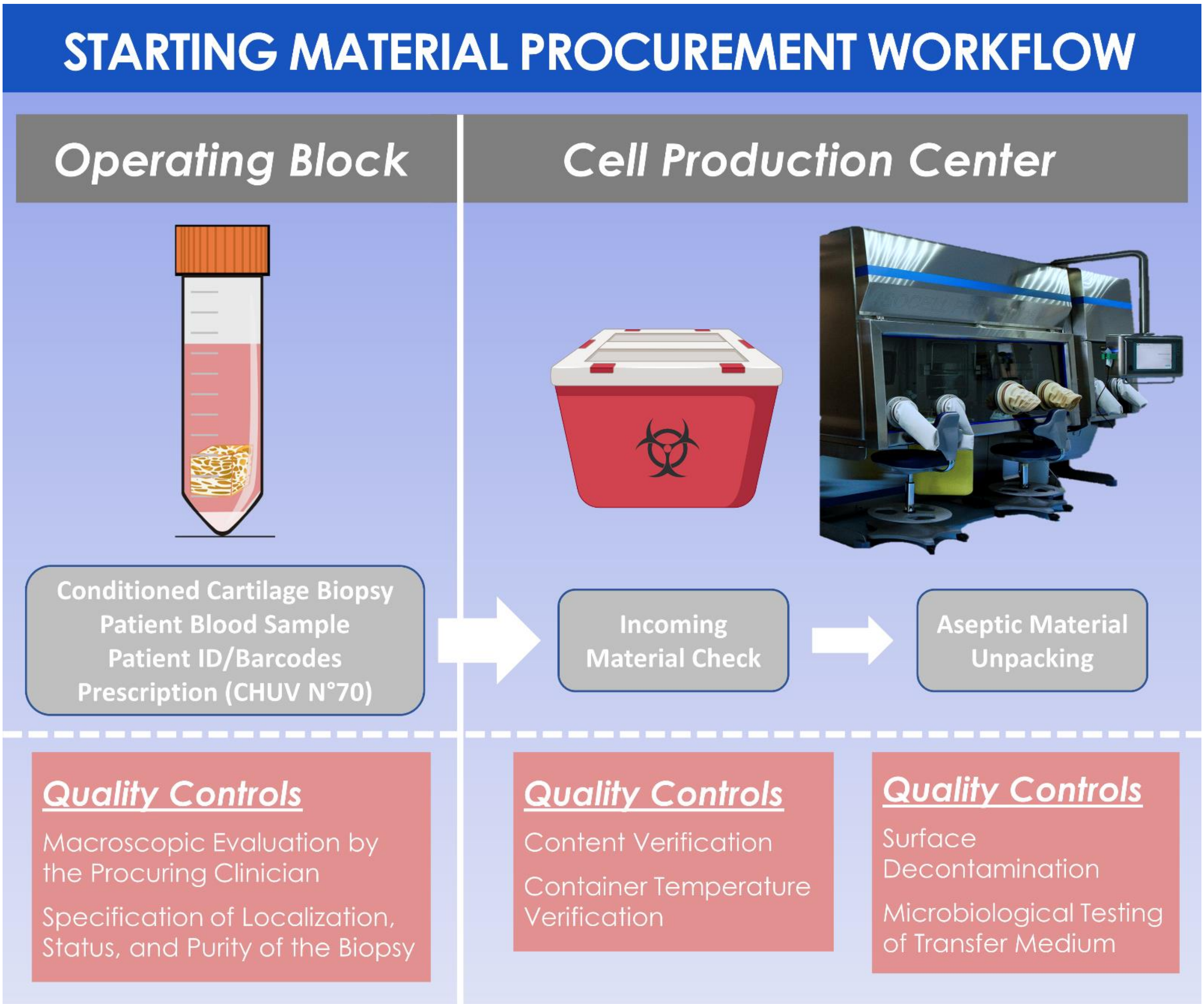

Figure S2. Schematic and illustrative workflow of healthy cartilage tissue biopsy procurement within the GMP manufacture of HAC-based cytotherapeutic products for ACI. The cartilage tissue biopsy is systematically processed and procured by the same qualified surgeon by arthroscopy, following which the biopsy is certified as being conform. © CHUV-CPC. ACI, autologous chondrocyte implantation; CHUV, centre hospitalier universitaire vaudois; CPC, cell production center; GMP, good manufacturing practices; HAC, human articular chondrocyte.

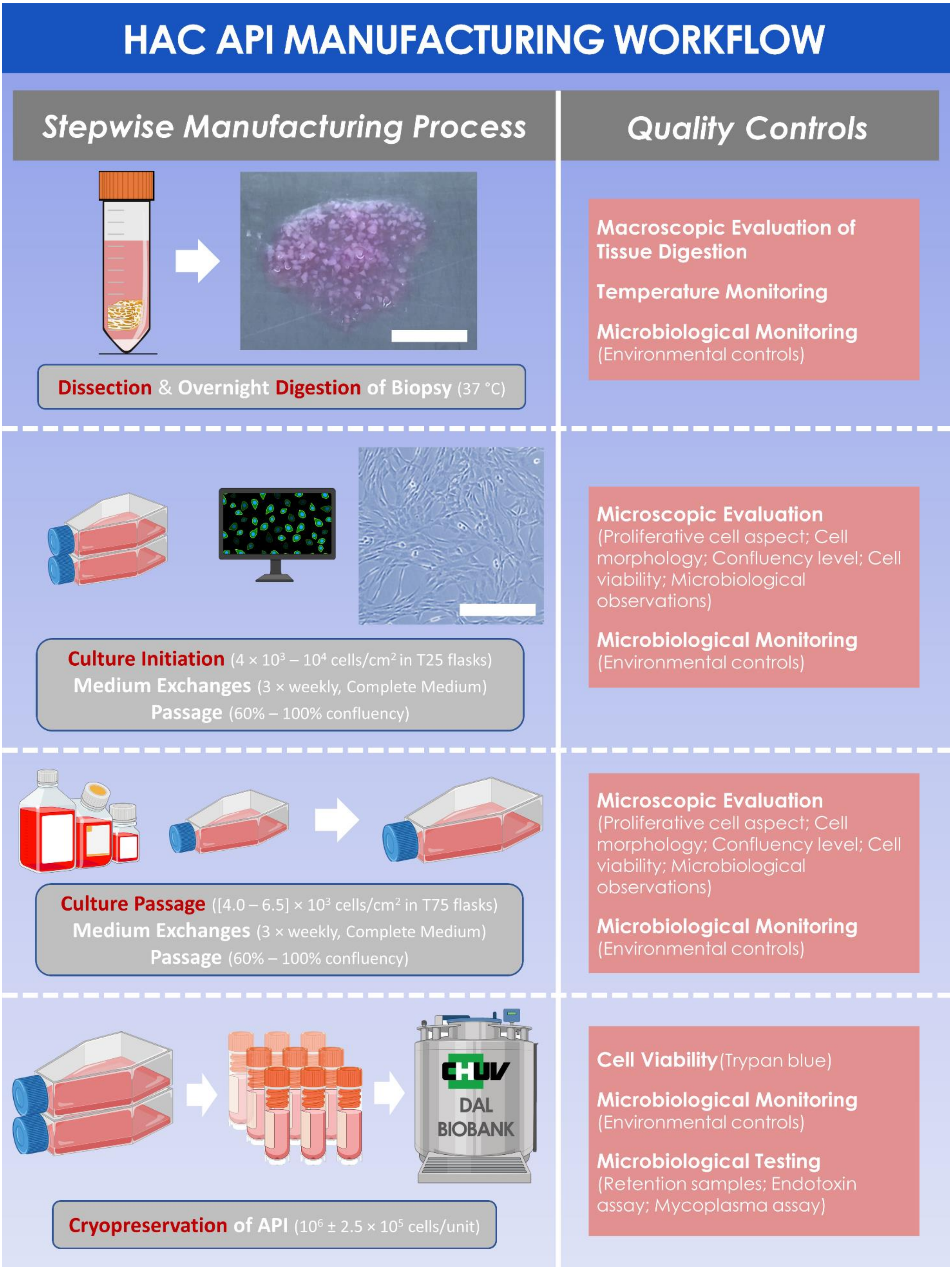

Figure S3. Schematic and illustrative workflow of HAC in vitro processing within the GMP manufacture of HAC-based cytotherapeutic products for ACI. Cryopreservation of the manufactured APIs is performed for several months before the finished product preparation and the eventual clinical implantation. Scale bars = 10 mm (cartilage biopsy fragments) or 100 µm (HAC culture). ACI, autologous chondrocyte implantation; API, active pharmaceutical ingredient; DAL, locomotor apparatus department; GMP, good manufacturing practices; HAC, human articular chondrocyte.

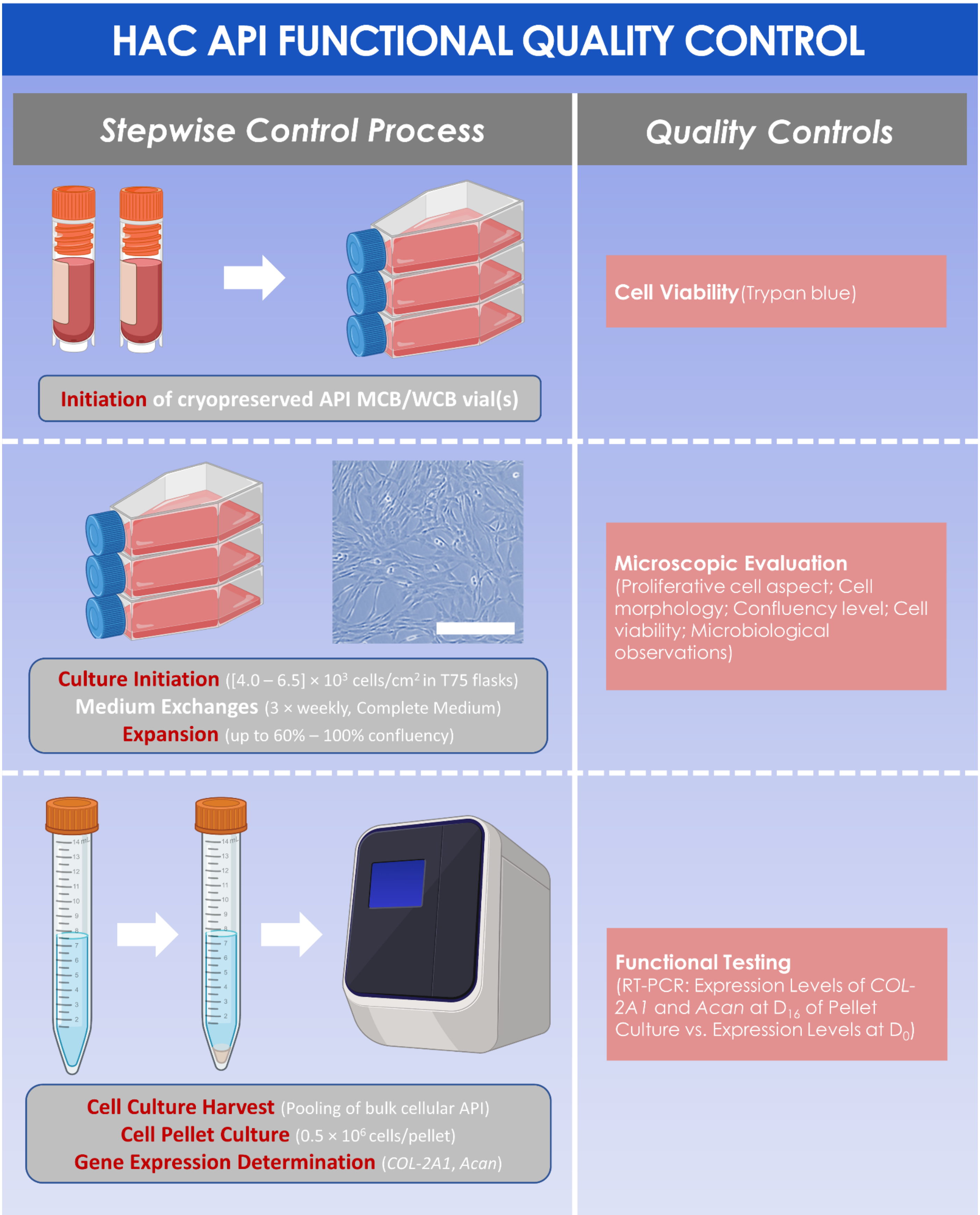

Figure S4. Schematic and illustrative workflow of HAC API functional quality control within the GMP manufacture of HAC-based cytotherapeutic products for ACI. Scale bar = 100 µm (HAC culture). ACI, autologous chondrocyte implantation; API, active pharmaceutical ingredient; GMP, good manufacturing practices; HAC, human articular chondrocyte; MCB, master cell bank; RT-PCR, real-time polymerase chain reaction; WCB, working cell bank.

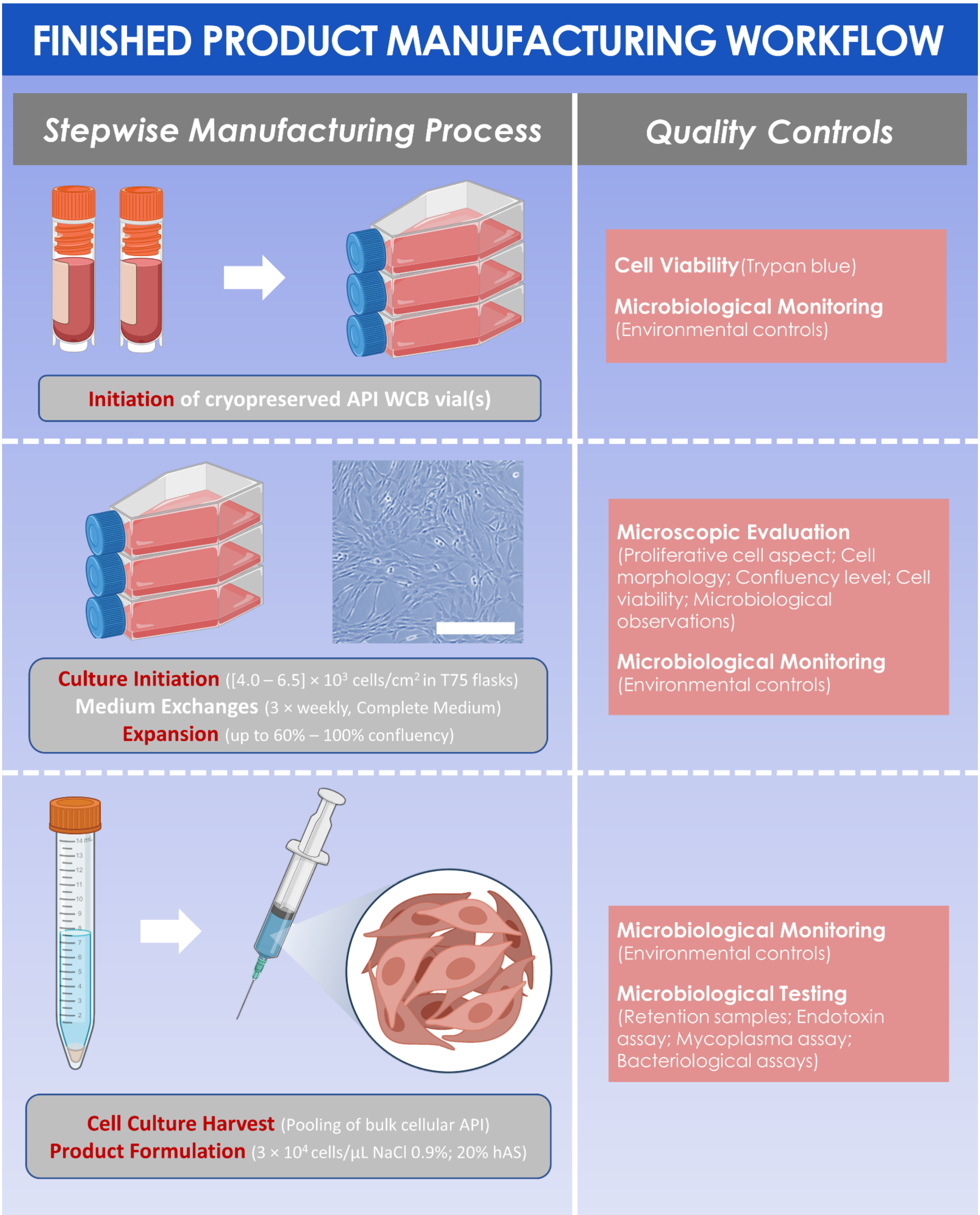

Figure S5. Schematic and illustrative workflow of the finished product preparation within the GMP manufacture of HAC-based cytotherapeutic products for ACI. The final product dose is of 2 × 10^6 cells/cm^2 of chondral or osteochondral lesion. Scale bar = 100 µm (HAC culture). ACI, autologous chondrocyte implantation; API, active pharmaceutical ingredient; GMP, good manufacturing practices; hAS, human autologous serum; WCB, working cell bank.
