# Supplementary material for: Retrospective Analysis of Autologous Chondrocyte-Based Cytotherapy Production for Clinical Use: GMP Process-Based Manufacturing Optimization in a Swiss University Hospital
Source: Cells. 2022 Mar 17;11(6):1016. doi: 10.3390/cells11061016 (PMC8947208; doi:10.3390/cells11061016)
Supplement: Supplementary file 1 [file cells-11-01016-s001.zip › Supplementary Tables.pdf]

Supplementary Tables

Table S1. Overview of autologous chondrocyte-based cytotherapeutic products used in clinical settings, following registration and commercialization in Europe, in Switzerland, and/or in North America. It is to note that, in addition to the products listed in the table, the product Chondrosphere® from co.don AG is approved in Germany (i.e., joints other than the knee). Regarding MACI®, the procedure was approved in Europe in 2013 but the authorization was not renewed in 2014. Another product, ChondroCelect® from TiGenix NV was authorized in Europe from 2009 to 2016. DMEM, Dulbecco's modified Eagle medium; EMA, European Medicines Agency; FDA, Food and Drug Administration; HAC, human articular chondrocytes; ICRS, International Cartilage Regeneration and Joint Preservation Society; NA, non-applicable or information unavailable.

Product Description / Trade Name	Identified Manufacturer	Authorization Type	Indication / Chondral Lesion Type [1]	Biopsy Zone	Biopsy Size (mg)	Lesion Size to Treat (cm^2)	Product / Procedure Specificities
**NOVOCART® 3D**<br>Cells/type I and III collagen scaffold	Tetec AG	Germany (2014)<br>Switzerland (2014)<br><br>EMA and FDA approval (ongoing)	Grade III/IV	Femoral condyle (non-loaded zone)	≈ 200	2–17	• Quantity of blood drawn: 140 mL<br>• Enzymatic digestion: NA<br>• Culture media: NA, with autologous serum<br>• Culture duration: 3–4 weeks<br>• Cryopreservation of cells: possible
**CaRes®**<br>Cells/type I collagen scaffold	Arthro-Kinetics AG	Some European countries, Turkey, Iran, and China	Grade III/IV	Femoral condyle (non-loaded zone)	150–250	3.5–14	• Quantity of blood drawn: 120–140 mL<br>• Enzymatic digestion: collagenase<br>• Cells non-amplified, directly mixed after isolation with collagen type I (rat) gel (3 mg/mL) and 20% autologous serum<br>• Culture duration: 2 weeks<br>• Cryopreservation of cells: not possible
**Spherox®**<br>Cell aggregates	co.don AG	EMA (2017)<br>Switzerland (2019)	Grade III/IV	Femoral condyle (non-loaded zone)	NA	≤ 10	• Enzymatic digestion: NA<br>• Culture media: NA, with autologous serum<br>• Culture duration: 3–4 weeks<br>• Cryopreservation of cells: not possible
**BioSeed®-C**<br>Cell suspension	Biotissue AG	Some European countries	Grade III/IV	Femoral condyle (non-loaded zone)	NA	≤ 10	• Enzymatic digestion: NA<br>• Culture media: NA, with autologous serum<br>• Culture duration: 3–4 weeks<br>• Cryopreservation of cells: possible

**MACI®**<br>Cells/type I and III collagen scaffold	Vericel Corporation	FDA (2016)	Grade III/IV	Femoral condyle (non-loaded zone)	≈ 200	3–20	• Enzymatic digestion: NA<br>• Culture media: NA, with autologous serum<br>• Culture duration: 3–4 weeks<br>• Cryopreservation of cells: possible

[1] According to the ICRS classification.

Table S2. General risk analysis matrix established for the assessment of the sourcing, procurement, and GMP culture initiation of primary HAC cell types in view of therapeutic ACI applications. ACI, autologous chondrocyte implantation; API, active pharmaceutical ingredient; GMP, good manufacturing practices; HAC, human articular chondrocyte; QC, quality control.

**Parameters**	**Pre-Mitigation**			**Risk Severity** (0–3) [1]	**Risk Likelihood** (0–2) [2]	**Risk Level** (0–2) [3]	**Mitigations**	**Post-Mitigation Risk Level** (0–2) [4]
	**• Risk**	**• Cause**	**• Effects**					
**Donor Qualification**	• Seropositivity for specified pathogens<br>• Seropositivity for unspecified pathogens<br>• Presence of specified exclusion criteria	• Inadequate anamnesis<br>• Inadequate testing scheme<br>• Presence of undetectable or latent infection	• Donor qualification failure<br>• Production of contaminated API<br>• API qualification failure	**3**	**1**	**2**	• Thorough patient anamnesis<br>• Use of specified inclusion and exclusion criteria<br>• Use of highly specific and sensitive donor screening methods	**0**
**Biopsy Qualification**	• Anatomical or physiological abnormality<br>• Insufficient biopsy size	• Inadequate anamnesis<br>• Inadequate biopsy harvest	• Biopsy qualification failure	**2**	**1**	**2**	• Extensive patient screening and anamnesis<br>• Biopsy harvest by qualified and experienced surgeon	**0**
**Cell Type Instability**	• Non-qualification for in vitro culture<br>• Apparition of tumorigenicity/toxicity	• Spontaneous mutation<br>• Cells beyond acceptable in vitro age<br>• Non-adaptation to in vitro culture	• Critical sustainability problematic<br>• Critical safety problematic	**3**	**1**	**2**	• Use of cells at low in vitro passage levels<br>• Qualification of cells for in vitro serial expansion	**0**

**Low Potential for Cell Banking**	• Low cell resistance to cryopreservation	• High cell sensitivity to cryogenic shock	• Critical quality problematic<br>• Critical sustainability problematic	**2**	**1**	**2**	• Monitoring of cell culture quality parameters<br>• Qualification and validation of materials and consumables	**0**
**Contamination of Biological Materials & Cell Banks**	• Introduction of extraneous contaminants by reagents, equipment, material, personnel<br>• Emergence of latent or transient virus<br>• Cross-contamination by a similar cell strain<br>• Cell population switch	• Adventitious agent introduction during manufacture, transport, or storage<br>• Inadequate segregation of cultures<br>• Poor initial population purity<br>• Inadequate manufacturing process<br>• Insufficient characterization of cell type	• Inadequate cell type in manufactured batch<br>• Contamination of manufactured batch<br>• Critical quality problematic	**3**	**1**	**2**	• Aseptic biological material procurement environment<br>• Class A manufacturing environment<br>• Selection of qualified and tested materials and reagents<br>• Environmental controls during open-container manipulations<br>• Minimization of open-container processes<br>• Minimization of contact processes<br>• Use of sterile single-use consumables<br>• Retention sample testing<br>• Post-production testing and batch qualification<br>• Identity and purity QCs of cultured cell populations	**0**

[1] The risk severity is classified as (0) or "acceptable", as (1) or "tolerable", as (2) or "undesirable", or as (3) or "intolerable". [2] The risk likelihood is classified as (0) or "improbable", as (1) or "possible", or as (2) or "probable". [3] The risk level is classified as (0) or "low", as (1) or "medium", or as (2) or "high". [4] The post-mitigation risk level is classified as (0) or "low", as (1) or "medium", or as (2) or "high".

Table S3. General risk analysis matrix established for the assessment of the in vitro GMP cell banking of primary HAC cell types in view of therapeutic ACI applications. ACI, autologous chondrocyte implantation; API, active pharmaceutical ingredient; GMP, good manufacturing practices; HAC, human articular chondrocyte; MCB, master cell bank; QC, quality control; WCB, working cell bank.

**Parameters**	**Pre-Mitigation**			**Risk Severity (0–3)** [1]	**Risk Likelihood (0–2)** [2]	**Risk Level (0–2)** [3]	**Mitigations**	**Post-Mitigation Risk Level (0–2)** [4]
	**• Risk**	**• Cause**	**• Effects**					
**Cell Viability**	• Loss of cell viability	• Inadequate storage or handling	• Reduction of cell manufacturing yield<br>• Reduced cell batch quality	**2**	**1**	**2**	• Storage temperature stability validation and monitoring<br>• Iterative total and viable cell enumeration<br>• Monitoring of cell culture quality [5]<br>• Rinsing of detached cells in culture	**0**
**Cell Bank Storage System Failure**	• Critical rise in vial temperature/vial thawing<br>• Catastrophic defect in vial structure or in Dewar storage tank system [6]	• Material and equipment failures<br>• System failures<br>• Absence of storage system or cooling liquid replenishing system redundancies	• Loss of vial batch or loss of whole cell banks	**3**	**0**	**1**	• Use of qualified primary containers (e.g., polymeric vials) and storage tanks (e.g., on-line or off-line tanks)<br>• Segregation of high-value vials in redundant storage systems/storage facilities<br>• Segregation of high-value vials in redundant storage tanks<br>• Nitrogen level/temperature monitoring and alarms<br>• Critical failure alarms<br>• Regular inspection of storage tanks<br>• Inspection of individual vials at the time of cell initiation	**0**

**Cross-Contamination or Cell Population Switch**	• Cross-contamination by a similar cell strain<br>• Cell population switch	• Inadequate segregation of cell cultures<br>• Poor initial cell population purity	• Inadequate cell type introduced during manufacturing	**3**	**1**	**2**	• Iterative identity and purity QCs<br>• Segregation of cell strains to specific manufacturing areas and equipment<br>• Use of sterile single-use consumables	**0**
**Functional Loss of API**	• Ineffective product manufacture	• Inadequate cell manufacture or storage<br>• Poor cell type functional quality	• Rejection of finished product<br>• Ineffective therapeutic intervention	**3**	**1**	**1**	• Monitoring of cell culture quality<br>• Use of qualified and consistent in vitro cell passage levels<br>• Standard functional QCs	**0**
**Adventitious Contamination of MCBs**	• Contaminated MCB<br>• Non-qualification and rejection of MCB	• Adventitious agent introduction during manufacture, transport, or storage	• Loss of large quantities of cells<br>• Need for cell type re-establishment from new biopsy	**3**	**1**	**2**	• Class A manufacturing environment<br>• Environmental controls during open-container manipulations<br>• Selection of qualified and tested materials and reagents<br>• Minimization of open-container processes<br>• Minimization of contact processes<br>• Use of sterile single-use consumables<br>• Retention sample testing<br>• Post-production MCB qualification	**0**

**Adventitious Contamination of WCBs**	• Contaminated WCB<br>• Non-qualification and rejection of WCB	• Adventitious agent introduction during manufacture, transport, or storage	• Loss of cell batch<br>• Need for WCB re-establishment from MCB	**2**	**1**	**2**	• Qualification of source MCBs<br>• Class A manufacturing environment<br>• Environmental controls during open-container manipulations<br>• Selection of qualified and tested materials and reagents<br>• Minimization of open-container processes<br>• Minimization of contact processes<br>• Use of sterile single-use consumables<br>• Retention sample testing<br>• Post-production WCB qualification	**0**
**Cellular API Tumorigenicity**	• Tumoral proliferation of biological API	• Spontaneous mutation<br>• Cells beyond acceptable in vitro age	• Tumor formation	**3**	**0**	**1**	• Use of autologous non-engineered cells<br>• Use of cells at low in vitro passage levels	**0**

[1] The risk severity is classified as (0) or "acceptable", as (1) or "tolerable", as (2) or "undesirable", or as (3) or "intolerable". [2] The risk likelihood is classified as (0) or "improbable", as (1) or "possible", or as (2) or "probable". [3] The risk level is classified as (0) or "low", as (1) or "medium", or as (2) or "high". [4] The post-mitigation risk level is classified as (0) or "low", as (1) or "medium", or as (2) or "high". [5] Monitoring includes proliferative cellular morphology, cell adhesion, growth rate, confluency level, cell monolayer homogeneity, sub-population exclusion, and gross microbiological contamination exclusion. [6] Includes rupture or explosion of vials and catastrophic defect in liquid nitrogen auto-filling system.

Table S4. Specific risk analysis matrix established for the assessment of the microbiological safety (i.e., excluding viruses) of primary HAC cell types, considering the cells as cryopreserved APIs for medicinal products. The microbiological safety (i.e., the absence of bacteria, fungi, mycoplasma, endotoxins) of the materials serving for the GMP manufacture of medicinal products is appropriately insured at the time of the selection of starting, raw, and ancillary materials and testing thereof, during production, and during post-production testing. API, active pharmaceutical ingredient; GMP, good manufacturing practices; HAC, human articular chondrocytes; MCB, master cell bank; QC, quality control; WCB, working cell bank.

**Parameters**	**Pre-Mitigation**			**Risk Severity** (0–3) [1]	**Risk Likelihood** (0–2) [2]	**Risk Level** (0–2) [3]	**Mitigations**	**Post-Mitigation Risk Level** (0–2) [4]
	**• Risk**	**• Cause**	**• Effects**					
**Species of Origin**	• Risk of infection by zoonotic pathogens	• Inclusion of infected donor materials	• Zoonotic contamination of API and infection of patient	**3**	**0**	**1**	• Selection of human starting materials<br>• Thorough testing for pathogens with human tropism	**0**
**Tissue of Origin**	• Use of contaminated starting materials	• Use of tissue type prone to contamination	• Contamination of the API<br>• Infectious risk for the patient	**3**	**1**	**1**	• Selection of tissue with low probability of high contaminant yield<br>• Thorough qualification of donor<br>• Thorough qualification of biopsy	**0**

**Contamination During API Manufacturing**	• Introduction of extraneous contaminant by reagents, equipment, material, personnel<br>• Emergence of latent or transient contaminant in culture	• Inadequate manufacturing process<br>• Inadequate control process<br>• Insufficient initial characterization of cell type<br>• Absence of purification regimen and terminal sterilization	• Contamination of the API<br>• Infectious risk for the patient	**3**	**1**	**2**	• Qualification of source cell banks<br>• Class A manufacturing environment<br>• Selection of qualified and tested materials and reagents<br>• Environmental controls during open-container manipulations<br>• Minimization of open-container processes<br>• Minimization of contact processes<br>• Use of sterile single-use consumables<br>• Retention sample testing<br>• Post-production cell bank testing and qualification<br>• Post-production bulk product and final product testing and qualification	**0**

**Infectivity or Iatrogenesis of Contaminated Cellular API**	• Iatrogenic infection of patient<br>• Inadequate management of patient pathology	• Non-functional or potentially iatrogenic API	• Patient contamination<br>• No amelioration or worsening or patient health status	3	1	2	• Qualification of MCBs/WCBs<br>• Class A manufacturing environment for API<br>• Environmental controls during open-container manipulations<br>• Retention sample testing<br>• Post-production API testing and qualification<br>• Post-production bulk product and final product testing and qualification	0
**Amount of API per Product Dose**	• Contamination of patient with large dose of pathogen	• Large dose of API per product dose	• Higher susceptibility toward infection and severe consequences	3	1	1	• Use of relatively small API quantity per product dose<br>• Use of sensitive detection methods for specified contaminants during testing<br>• Use of restrictive pathogen limits and thresholds	0
**Process Controls (donor, starting material, products)**	• Failure in implemented process controls<br>• Inadequacy of process controls	• Systemic error in implemented controls<br>• Occasional error in implemented controls<br>• Apparition of new unspecified contaminants	• Liberation of contaminated API batch<br>• Infectious risk for the patient	3	1	2	• Iterative update of process controls<br>• Iterative validation of process controls<br>• Redundant process controls<br>• Process controls implemented at the appropriate stages of GMP manufacture	0

**Biosafety Testing Scheme**	• Emergence of pathogen undetected in preliminary subcultures	• Presence of pathogen in undetectable quantities in cell seed	• Contamination of API batch<br>• Infectious risk for the patient	**3**	**1**	**1**	• Iterative update of testing schemes<br>• Iterative and redundant testing steps<br>• Full microbiological quality testing of MCBs/WCBs	**0**

[1] The risk severity is classified as (0) or "acceptable", as (1) or "tolerable", as (2) or "undesirable", or as (3) or "intolerable". [2] The risk likelihood is classified as (0) or "improbable", as (1) or "possible", or as (2) or "probable". [3] The risk level is classified as (0) or "low", as (1) or "medium", or as (2) or "high". [4] The post-mitigation risk level is classified as (0) or "low", as (1) or "medium", or as (2) or "high".

Table S5. General risk analysis matrix established for HAC-based injectable finished products, adapted from the EMA Guideline EMEA/CHMP/410869/2006 "Guideline on human cell-based medicinal products". The specified parameters were established based on API and on the finished product specifications and on the critical quality attributes. API, active pharmaceutical ingredient; EMA, European Medicines Agency; HAC, human articular chondrocyte; QC, quality control; WCB, working cell bank.

Parameters	Pre-Mitigation			Risk Severity (0–3) [1]	Risk Likelihood (0–2) [2]	Risk Level (0–2) [3]	Mitigations	Post-Mitigation Risk Level (0–2) [4]
	• Risk	• Cause	• Effects					
API Immunogenicity	• Immune reaction of recipient to API	• Immune recognition of cellular API by recipient organism	• Treatment failure<br>• Iatrogenesis	**3**	**1**	**1**	• Use of autologous cells as APIs<br>• No pooling of starting materials from distinct donors<br>• Exclusion of recipients with immunological/allergic risk factors	**0**
Low Cell Viability in Final Product	• Insufficient product efficacy in case of low cell viability	• Inadequacy of product formulation<br>• Inadequacy of product storage and handling<br>• Inadequacy of product administration	• Treatment failure	**2**	**1**	**1**	• Qualification and validation of finished product formula<br>• Validation of cell viability maintenance following product reconstitution, transport, and administration (i.e., full validity period)<br>• Viability determination QC at the time of finished product reconstitution<br>• Short product validity period<br>• Appropriate specified product transport and administration modalities	**0**

**Level of API Manipulation**	• Mutagenicity, oncogenicity, or tumorigenicity of API	• High manipulation of cells<br>• Extensive in vitro cell culture	• Formation of tumors in patients	**3**	**0**	**1**	• No genetic manipulation of cells<br>• No immortalization of cells<br>• No use of viral tools for cell manufacture<br>• Use of cells at low passage levels	**0**
**Adventitious Contamination during Finished Product Manufacturing**	• Introduction of extraneous contaminants by reagents, equipment, material, personnel	• Inadequate manufacturing process (i.e., including storage and transport)<br>• Inadequate reagents, materials<br>• Inadequate control process<br>• Presence of latent virus in materials<br>• Absence of purification regimen and terminal sterilization	• Contamination of product<br>• Infectious risk for the patient	**3**	**1**	**2**	• Testing and qualification of API MCB/WCB<br>• Class A manufacturing environment<br>• Selection of qualified and tested materials and reagents<br>• Environmental controls during open-container manipulations<br>• Minimization of open-container processes<br>• Minimization of contact processes<br>• Use of sterile single-use consumables<br>• Retention sample testing<br>• Post-production final product testing and qualification	**0**
**Product Mode of Administration**	• Systemic exposure to injectable product	• Incorrect product administration site<br>• Systemic distribution of APIs	• Systemic effects of APIs	**1**	**1**	**1**	• Robust qualification and experience of orthopedic surgeon<br>• Historical clinical safety data present<br>• No evidence of high dose-related adverse effects	**0**

**Combination Product**	• Incompatibility between API and vehicle<br>• Formation of toxic degradation products	• Biological or chemical incompatibility or reaction between API and vehicle	• Treatment failure<br>• Iatrogenesis	**3**	**0**	**1**	• Qualification of vehicle<br>• Qualification of finished combination product<br>• Historical clinical safety data present with finished product	**0**
**Duration of Exposure to Product**	• Chronic toxicity of product	• Extensive and repeated exposure to product applied on wounded tissues	• Treatment failure<br>• Iatrogenesis	**3**	**0**	**1**	• Physiological product clearance<br>• Limited number of product applications<br>• Limited persistence of product/API in wounded tissues	**0**
**Availability of Clinical Safety Data and Experience**	• Insufficient safety data/experience gathered	• No clinical recording of historical product use	• Absence of tangible evidence for retrospective safety evaluation of product	**2**	**0**	**0**	• Several years of safe clinical use<br>• Multiple peer-reviewed scientific publications on safety and efficacy of API/products<br>• Prospective and retrospective clinical trials performed for multiple similar indications	**0**

[1] The risk severity is classified as (0) or "acceptable", as (1) or "tolerable", as (2) or "undesirable", or as (3) or "intolerable". [2] The risk likelihood is classified as (0) or "improbable", as (1) or "possible", or as (2) or "probable". [3] The risk level is classified as (0) or "low", as (1) or "medium", or as (2) or "high". [4] The post-mitigation risk level is classified as (0) or "low", as (1) or "medium", or as (2) or "high".
